# Mathematical modeling of COVID-19 in 14.8 million individuals in Bahia, Brazil

**DOI:** 10.1038/s41467-020-19798-3

**Published:** 2021-01-12

**Authors:** Juliane F. Oliveira, Daniel C. P. Jorge, Rafael V. Veiga, Moreno S. Rodrigues, Matheus F. Torquato, Nivea B. da Silva, Rosemeire L. Fiaccone, Luciana L. Cardim, Felipe A. C. Pereira, Caio P. de Castro, Aureliano S. S. Paiva, Alan A. S. Amad, Ernesto A. B. F. Lima, Diego S. Souza, Suani T. R. Pinho, Pablo Ivan P. Ramos, Roberto F. S. Andrade

**Affiliations:** 1 Center of Data and Knowledge Integration for Health (CIDACS), Instituto Gonçalo Moniz, Fundação Oswaldo Cruz, Salvador, Bahia Brazil; 2 Centre of Mathematics of the University of Porto (CMUP), Department of Mathematics, Porto, Portugal; 3 Instituto de Física, Universidade Federal da Bahia, Salvador, Bahia Brazil; 4 Fundação Oswaldo Cruz, Porto Velho, Rondônia, Brazil; 5 College of Engineering, Swansea University, Swansea, Wales UK; 6 Instituto de Matemática e Estatística, Universidade Federal da Bahia, Salvador, Bahia Brazil; 7 Instituto de Física, Universidade de São Paulo, São Paulo, Brazil; 8 Oden Institute for Computational Engineering and Sciences, The University of Texas at Austin, Austin, TX USA

**Keywords:** Computational models, SARS-CoV-2, Infectious diseases, Epidemiology

## Abstract

COVID-19 is affecting healthcare resources worldwide, with lower and middle-income countries being particularly disadvantaged to mitigate the challenges imposed by the disease, including the availability of a sufficient number of infirmary/ICU hospital beds, ventilators, and medical supplies. Here, we use mathematical modelling to study the dynamics of COVID-19 in Bahia, a state in northeastern Brazil, considering the influences of asymptomatic/non-detected cases, hospitalizations, and mortality. The impacts of policies on the transmission rate were also examined. Our results underscore the difficulties in maintaining a fully operational health infrastructure amidst the pandemic. Lowering the transmission rate is paramount to this objective, but current local efforts, leading to a 36% decrease, remain insufficient to prevent systemic collapse at peak demand, which could be accomplished using periodic interventions. Non-detected cases contribute to a ∽55% increase in *R*_0_. Finally, we discuss our results in light of epidemiological data that became available after the initial analyses.

## Abstract (web summary)

Low-resource settings can face additional challenges in managing the COVID-19 pandemic. Here, the authors use mathematical modelling to investigate transmission in the state of Bahia, Brazil, and quantify control measures needed to prevent the hospital system becoming overwhelmed.

## Introduction

In December 2019, clusters of a respiratory disease attributed to a potentially novel coronavirus were identified. This hypothesis was rapidly confirmed, and the virus was named as severe acute respiratory syndrome coronavirus 2 (SARS-CoV-2), the causal agent of coronavirus disease 2019 (COVID-19). This novel coronavirus rapidly spread across Asia, Europe, and other continents, achieving pandemic status, as determined by the World Health Organization, on March 11, 2020. As of October 2020, all parts of the world were, to varying degrees, impacted by the COVID-19 epidemic, with more than 40 million cases and 1.1 million deaths reported globally^1^, in what can be construed as the worst pandemic since the Spanish flu (1918–1920).

A prominent feature of the current pandemic is the high person-to-person transmissibility of the virus, with a basic reproduction number (*R*_0_) estimated at 2.2–2.5 in Wuhan, China, where initial cases were detected^2,3^. Other worrying aspects are the severity of clinical complications and the lack of vaccines or effective drugs to, respectively, prevent disease and accelerate the patient’s recovery. Consequently, the only effective mechanisms currently available to dampen viral spread are non-pharmaceutical interventions (NPI), and the population’s adherence thereof. Among these are social isolation and distancing, quarantine, travel restrictions, and changes in individual behavior, such as the widespread usage of face masks and heightened preoccupation with hygiene^4^.

The sudden increase in demand for hospitalization during the pandemic, leading to collapse in health systems due to insufficient medical infrastructure and healthcare resources, has particularly impacted countries with limited healthcare infrastructure, such as those in Latin America. Brazil, the largest country in this region, provides a cautionary example of the profound impacts of COVID-19 on health systems. The first confirmed case of COVID-19 in Brazil occurred on February 26, 2020, in the state of São Paulo, although multiple independent introductions have occurred, fueled by returning international travelers^5^, and nationwide community transmission was declared on March 20. As of October, 2020, the number of confirmed COVID-19 cases exceeded 5.2 million nationally, with over 150,000 deaths reported. Bahia, located in northeast Brazil, has a population of 14.8 million throughout its 417 municipalities, with a territorial extension of 567,295 km^2^, comparable to that of France. In spite of its economic importance (with the sixth highest gross domestic product among all Brazilian states), the state of Bahia presents marked intra-regional disparities in terms of access to health, with hospitals and healthcare investments unequally distributed around the state^6^. Thus, Bahia is a representative example of how COVID-19 impacts health resources in low and middle-income countries, and the effects of measures implemented in an attempt to mitigate damaging consequences.

Mathematical models are proving instrumental in studying the current COVID-19 pandemic^7^, as well as in driving governmental policy. A hallmark of the latter was the radical shift in actions of some governments defending “herd immunity” strategies, as models produced by the Imperial College London projected massive death tolls before reaching this objective^8^. Substantial insights into the dynamics of disease spread can be gained by using compartmentalized models, such as 3-compartment SIR (susceptible-infected-recovered)^9^. Models that build on these principles have flourished in the recent literature, even extending the number of compartments to study other key aspects of COVID-19, including the role of asymptomatic transmission^10,11^, social distancing, and quarantine strategies^3,12–15^, as well as post-epidemic scenarios, e.g. the probability of novel outbreaks^16,17^. The need for hospitalization under various conditions has also been evaluated using mathematical modeling^18–20^.

In this work, we further explore hospitalization needs in a low-resource state during the COVID-19 pandemic, with particular emphasis given to hospital ward (throughout the text referred to as clinical) and intensive care unit (ICU) bed requirements. Particularly, we describe an 8-compartment model with variable disease transmissibility over time, considering transmission by asymptomatic/mild cases, which usually go undetected, hospitalization of severe cases (requiring clinical/ICU beds) and mortality. The parameters of this model are partially locally informed using data from hospitals dedicated to treating COVID-19 patients in the region, and partly calibrated against the data (cases, deaths) provided by local health authorities, with optimal parameters identified using particle swarm optimization metaheuristics. This model was applied to study the ongoing COVID-19 outbreak in the state of Bahia, Brazil, an example of a low-resource setting with pronounced inequalities in healthcare access, but could be extended and is directly applicable to other regions, offering the potential to aid in setting targets that may guide to the analysis of the evolving COVID-19 pandemic, in addition to informing the extent of governmental measures required. Finally, we performed an ex-post evaluation of the COVID-19 epidemic in Bahia using data that became available after the initial analysis, focusing on the actual clinical/ICU beds usage during the period, the number of COVID-19 cases and deaths, and the utility of the proposed model to describe the epidemic in real-time.

## Results

### Model sensitivity analysis

We first conducted a sensitivity analysis to evaluate the most influential parameters of the model. Of note, the variance-based method used accounts for interactions among the model variables. These results revealed the factor that reduces the infectivity of the asymptomatic/non-detected, *δ*, to be among the most influential parameters to every model output during the whole period evaluated. Also, the transmission rate *β* was identified as exerting an important role in the model dynamics, as expected. Particularly, during the first 30 days *β*_0_ is the most important parameter in the system, as indicated by higher values of the total effect index (*S*_*T*_). After this period, the importance of *β*_0_ decreases as that of *β*_1_ increases, eventually superseding the former as the most important parameter in the system. For *H*, *U*, and *D*, the most influential parameter during the initial stages of the simulation (before day 15) is the proportion of symptomatic needing hospitalization or ICU, *h*, together with the transmission rate (Supplementary Figs. 1, 2). The full analysis is presented in Supplementary Note 2.

### Effects of social distancing and governmental interventions on disease transmissibility were observable shortly after onset

**Fig. 1:**
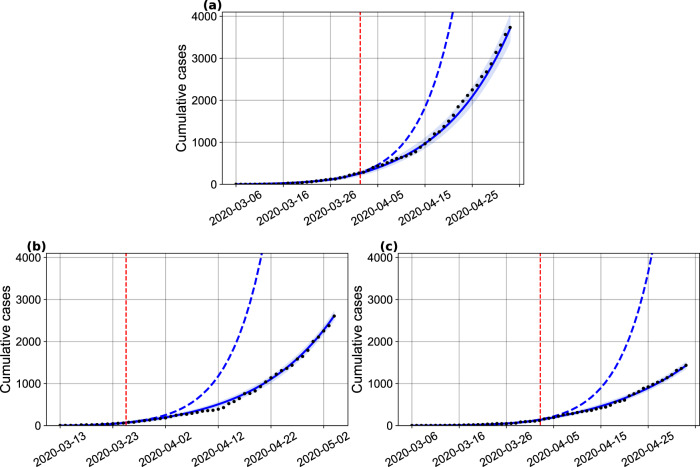
Projection of the the number of cases with a changing transmission rate. (**a**) in Bahia; (**b**) in Salvador, and (**c**) in the remaining 416 municipalities. The parameters *κ* = 1/4, *p* = 0.2, *γ*_*a*_ = 1/3.5, *γ*_*s*_ = 1/4 were fixed and *h* was set to zero for the capital and inland cities. The black dots correspond to the actual number of cases. The vertical dashed red lines are the dates of transition from *β*_0_ to *β*_1_. The blue dashed and full lines represent the evolution of the epidemic with a fixed transmission rate *β*_0_ and with both *β*_0_ and *β*_1_, respectively. The shaded error bands represent 95% confidence intervals of the mean calculated using the weighted non-parametric bootstrap method. Raw data from March 6 to May 4, 2020 are shown in this graph.

We started our analyzes by assessing the effects in disease transmissibility that local non-pharmaceutical interventions (NPI) have produced in the Bahia state, in its capital Salvador, as well as in the remaining cities (all municipalities in the state except the capital). For this, the model was fitted using the number of confirmed cases as declared by local authorities (Fig. 1) and we estimated parameters related to the transmission rate (*β*_0_, *β*_1_), the time point when it changes, and the factor that reduce the infectivity of the asymptomatic/non-detected, *δ*. We observed that the initial (pre-intervention) transmission rate was *β*_0_ = 1.28 ([1.26–1.30] 95% CI). A reduction of 36% on the transmission rate, yielding *β*_1_ = 0.92 ([0.91, 0.93] 95% CI), is evidenced around April 2 (27 days after the first confirmed case in the state). In Salvador (with a population of 2.6 million people), this reduction was of 54.7% (on March 26), while the remaining cities displayed a decrease of approximately 40.6% (on April 3, 2020). The factor that reduces the infectivity of the asymptomatic/non-detected was determined to be *δ* = 0.34 ([0.33, 0.35] 95% CI) at the state level, *δ* = 0.71 ([0.69, 0.72] 95% CI) in the capital and *δ* = 0.62 ([0.60, 0.64] 95% CI) in the remaining cities. The basic reproduction numbers for Bahia, Salvador and the other 416 municipalities were, respectively, of *R*_0_ = 2.25 ([2.19–2.31] 95% CI), 3.56 ([3.44–3.69] 95% CI) and 2.45 ([2.36–2.55] 95% CI).

**Fig. 2:**
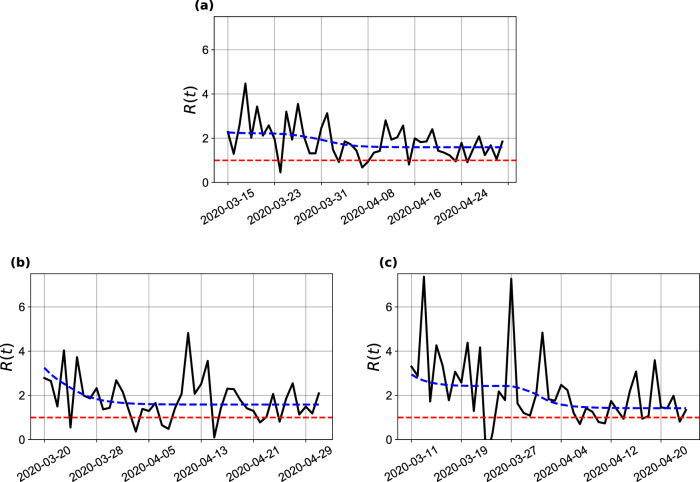
Effective reproduction number. Results for (**a**) Bahia, (**b**) Salvador, and (**c**) the remaining 416 municipalities up to May 4, 2020. The black solid lines represent the ${{\mathcal{R}}}_{t}$ calculated with reported number of new cases; the blue dashed lines represent the ${{\mathcal{R}}}_{t}$ calculated with the new number of simulated cases obtained from the model. The red dashed lines indicate ${{\mathcal{R}}}_{t}=1$.

These results show that the combined effects of changes in human behavior with the governmental policies of movement restriction resulted in significant decreases of the transmission rate, as measured by the *β* parameter. However, these efforts are still insufficient to curb the epidemic in the state, as the basic effective reproduction number still exceeds 1 (see Fig. 2), indicating a scenario of continuing growth. Next, we present the results of the consequence of this growth in local health resources.

### Projecting hospitalization requirements in Bahia, Brazil: challenges for low-resource settings

**Fig. 3:**
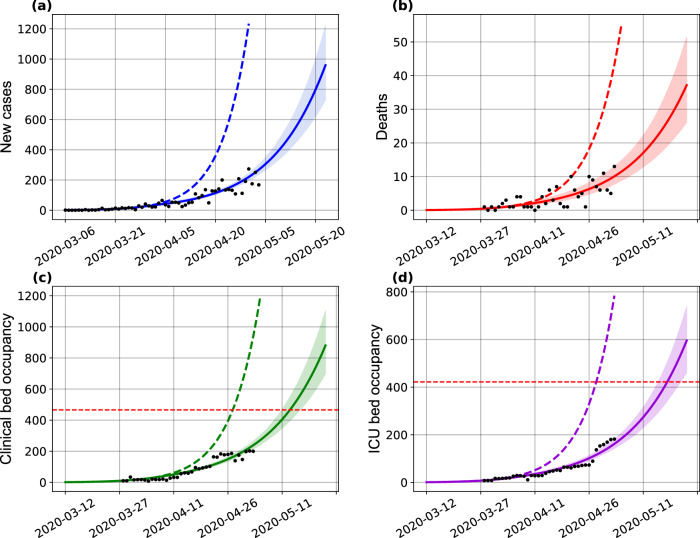
Effects of the implemented interventions in Bahia. Effects on the number of (**a**) cases, (**b**) deaths, (**c**) clinical hospitalization, and (**d**) ICU bed requirements at the state level. The horizontal red dashed lines are, respectively, the current capacity for beds for clinical hospitalization (466 beds) and ICUs (422 beds). The blue dashed and full lines represent the evolution of the epidemic with a fixed transmission rate *β*_0_ and with both *β*_0_ and *β*_1_, respectively. The shaded error bands represent 95% confidence intervals of the mean calculated using the weighted non-parametric bootstrap method. Residual analysis to visualize a tendency between the data and simulations are presented in Supplementary Fig. 7. The assumed parameter values are shown in Supplementary Table 3. Raw data from March 6 to May 4, 2020 are shown in this graph.

We evaluated the burden on hospitalization needs imposed by the COVID-19 epidemic at the state level, as well as the effects of NPI strategies on these requirements. We also estimated the total number of deaths projected by the model in the absence and during the enforcement of distancing measures. Our results, presented in Fig. 3, show that, in the absence of interventions, the state level availability of clinical beds would be exhausted by April 24, 2020. With the maintenance of the current level of interventions, this depletion is shifted in time and would occur by May 9. Analogously, the demand for ICU beds would exceed the installed capacity by April 26 in the absence of interventions, and by May 13 with the current rate of interventions.

The real-world, state-level data obtained for May 4, the last day with available bed occupancy observations, shows that 240 (51.5%) clinical beds were occupied, while 176 (41.7%) ICU beds were in use. Our model-based analysis yields an increase in these numbers by 6.5 (1581 beds) and 6.4 (1131 beds) times for clinical and ICU beds, respectively, if interventions had not been adopted in the state. On the other hand, measures enforced decreased the number of cases and deaths by 7 and 4 times, respectively, compared to the scenario where no measures are in place.

These results underscore the impact that the ongoing COVID-19 epidemic imposes on hospital resources and mortality, and particularly explicit the challenges faced by countries with more limited healthcare systems. Even if we consider overestimation of the prediction results, the simple doubling of the current real-world bed occupancy data would already result in exceeding the current availability of clinical and ICU beds in the state.

The obtained ${{\mathcal{R}}}_{0}$ is as before and the effective reproduction number is presented in Fig. 2. We can notice a trend of reduction on the effective reproduction number, although ${\mathcal{R}}(t)$ is above one throughout most of the time series for the state of Bahia. Interestingly, our analysis of how non-detected cases (asymptomatic/mild infections) influence the course and dynamic of transmission revealed that these individuals contribute to an increase of 55.03% on the basic reproduction number.

### A model-informed strategy of periodic interventions to reduce COVID-19 transmissibility in an effort to protect health systems

The previous results revealed the favorable effects that interventions resulting in decreased transmission rate have on shifting the peak of hospitalization saturation (complete occupancy of available beds), and in decreasing the number of cases and deaths. However, these results showed that complete saturation is inevitable under our local conditions. We next evaluated to what extent more vigorous restriction policies, and their duration/periodicity, would be useful in order to prevent the complete collapse of the state-level health system. To address this question, we used the SEIIHURD model to study the epidemic dynamics under various scenarios.

**Fig. 4:**
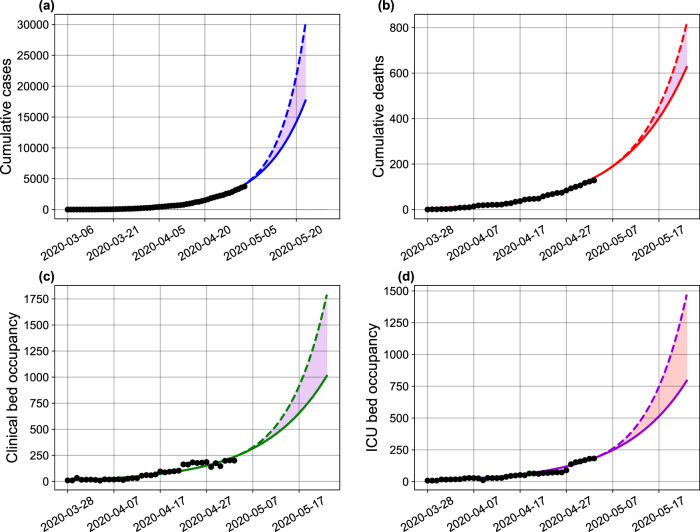
Effect of easing the social distancing for individuals with asymptomatic/mild infections in Bahia. Impact on the (**a**) number of cases, (**b**) deaths, (**c**) clinical hospitalization, and (**d**) ICU bed requirements at the state level. Here, the value of *δ* has been increased by 50% (*δ* = 0.51 in this simulation). The black dots correspond to the actual number of cases (**a**), deaths (**b**), and hospital bed occupancy (**c**)–(**d**). The assumed parameter values are shown in Supplementary Table 3. Raw data from March 6 to May 4, 2020 are shown in this graph.

Initially, and in order to assert that disease transmissibility is the driving factor leading to increased hospitalization requirements, we considered a scenario where the transmission rate of asymptomatic/non-detected individuals was increased by 50% starting in May 5 (Fig. 4). After 20 days, we noticed an increase of cumulative cases and deaths of, respectively, 50%, and 37%. Accordingly, clinical and ICU bed requirements increase by 75% and 87.5%, respectively. This scenario is illustrative of a situation where the movement restriction of individuals asymptomatic or having only mild symptoms (non-detected infections) is eased.

By accounting for non-detected cases, our modeling-based approach allowed the estimation of the infection fatality ratio (IFR), which considers deaths as a proportion of the total number of cases irrespective of severity or symptomatology. Our simulations of the SEIIHURD model yielded an overall IFR of 0.69% ([0.67, 0.71] 95% CI) for the state of Bahia, in line with preliminary findings from a literature meta-analysis that reported an estimated IFR of 0.68% ([0.52, 0.82] 95% CI), characterized by extensive heterogeneity across countries^21,22^. In addition, modeling allows the estimation of the overall fraction of infected in the population exposed to the virus, allowing to investigate whether decreases in the transmission rate are driven more by a decreased pool of susceptibles or as a result of effective control policies. We estimated that, by May 4, 2020, around 0.1% of the population had been infected (either symptomatic or asymptomatic/non-detected) (Supplementary Fig. 3), in line with seroprevalence studies for the region^23^, reinforcing the benefits of control policies to contain the viral spread, at least in the initial epidemic phase.

The previous results confirmed the importance of controlling disease transmissibility. Then, we turned to set targets that would allow for an increase in the protection of the healthcare system. These scenarios are illustrated in Table 1. We show that an intervention that reduces the transmission rate by 25%, enforced on May 2, 2020 (7 days before the predicted collapse of the system), for 30 days, would not yield significant improvements, resulting in a gain of only 2 days until clinical beds collapse, and 8 days until ICU bed capacity is exhausted. Similar results can be achieved by a more punctual (7 days period), but more vigorous intervention reducing transmission rate by 50% (Table 1). More interestingly, a delay of about 40 days for clinical and ICU bed exhaustion can be achieved in a scenario where a 50% reduction on the transmission is sustained for 30 days, or when a 75% reduction is endured for 14 days (Supplementary Fig. 4).

**Table 1:** Scenarios of an immediate intervention in May 2, 2020, with variations of the transmission rate and intervention length.

Percentage of transmission rate reduction	Intervention length (days)	Date of hospitalization beds collapse (delay, in days, compared to baseline scenario)	Date of ICU beds collapse (delay, in days, compared to baseline scenario)
	7	05/11/20 (2)	05/17/20 (4)
25%	14	05/11/20 (2)	05/20/20 (7)
	30	05/11/20 (2)	05/21/20 (8)
	7	05/15/20 (6)	05/21/20 (8)
50%	14	05/24/20 (15)	05/31/20 (18)
	30	06/17/20 (39)	06/23/20 (41)
	7	05/22/20 (13)	05/27/20 (14)
75%	14	06/08/20 (30)	06/13/20 (31)
	30	07/17/20 (69)	07/21/20 (69)

The timing of interventions is also crucial under our model. If a vigorous intervention is only adopted on the day when clinical bed occupancy reaches its maximum availability (on May 9), a time lag will be needed in order to allow for patient turnover in hospitals. Once occupancy is below the total availability, interventions can be continued or suspended. In the latter case, hospitalization requirements recommence to rise until reaching the health system’s capacity once again. Under this scenario, an intervention that reduces the transmission rate by 25% will not be enough to protect health resources, even if policies are maintained for long periods of time (Supplementary Fig. 5). Similar results are seen when we consider the reduction of the transmission rate by 50%, as shown in Table 2. Thus, harsher efforts to contain disease transmissibility, and for more extended periods, are necessary to allow for a full recovery of the healthcare system.

**Table 2:** Scenarios of critical interventions adopted exactly when total clinical beds availability is reached (05/09/20), with variations of the transmission rate and intervention length.

Percentage of transmission rate reduction	Intervention length (days)	Date when the hospitalization threshold is re-achieved (days)	Date of second hospitalization collapse (days of delay)	Date when the ICU threshold is re-achieved (days)	Date of second ICU collapse (days of delay)
	7	does not occur^a^	–	^b^	05/18/20 (5)
	14	does not occur^a^	–	^b^	05/26/20 (13)
50%	30	does not occur^a^	–	^b^	06/21/20 (39)
	60	06/17/20 (39)	07/30/20 (43)	^b^	08/04/20 (83)
	90	06/17/20 (39)	09/10/20 (86)	^b^	09/15/20 (125)
	7	does not occur^a^	–	^b^	05/25/20 (12)
	14	05/26/20 (17)	06/05/20 (10)	^b^	06/12/20 (30)
75%	30	05/26/20 (17)	07/17/20 (52)	^b^	07/21/20 (69)
	60	05/26/20 (17)	09/26/20 (123)	^b^	09/30/20 (140)

–, does not apply.

^a^Hospitalization is not reduced enough to reach the actual capacity.

^b^ICU did not collapsed.

**Fig. 5:**
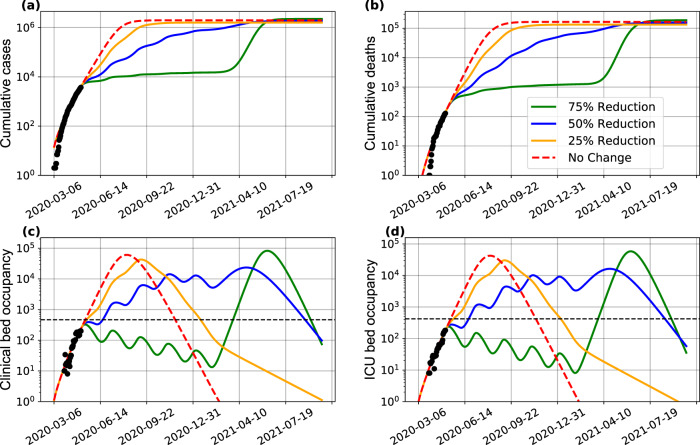
Effect of periodic interventions in Bahia. Simulated impact on the (**a**) number of cases, (**b**) deaths, (**c**) clinical hospitalization, and (**d**) ICU bed requirements at the state level. The transmission function *β*, as in Eq. (1), is defined by considering a reduction of 25% (yellow curves), 50% (blue curves), and 75% (green curves) on the *β*_1_ parameter. The red curves consider a scenario of no reduction in *β*_1_, and the period is the intervention window of 30 days. The dashed horizontal lines in (**c**) and (**d**) indicate the total number of clinical and ICU beds available in the state, respectively, in that moment. The black dots correspond to the actual number of cases (**a**), deaths (**b**), and hospital bed occupancy (**c**)–(**d**). The assumed parameter values are shown in Supplementary Table 3. Raw data from March 6 to May 4, 2020 are shown in this graph.

The previous results, combined, show that intense efforts to decrease COVID-19 transmissibility are needed in order to overcome a complete collapse of the healthcare system in a low-resource setting such as that encountered in Bahia, Brazil. Of note, under some of the presented scenarios full re-establishment of the hospitalization capacity may not be achieved if the timing to enforce more strict measures to decrease the transmission rate is not optimal. Accordingly, periodic interventions may be needed to control secondary waves of the outbreak. In Fig. 5 we illustrate the behavior of the spread dynamics of COVID-19 in Bahia if measures are periodically adopted. We present the behavior of implementing measures for a period of 30 days, followed by an easing of 30 days, and this being repeated periodically. These results show that even a reduction of 50% of the current transmission rate is insufficient to remain below the actual availability of health resources. Nevertheless, this implementation may be combined with the expanding of health capacities, limiting the need of more intense interventions.

### Real-time modeling

**Fig. 6:**
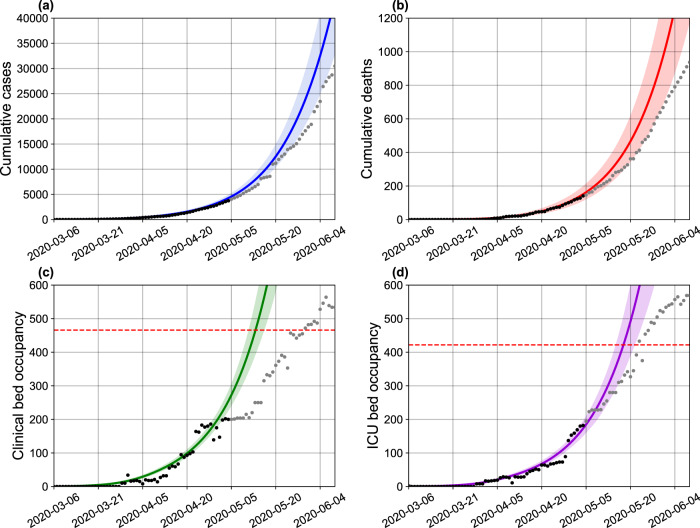
Real-time comparison between the modeling analysis in Bahia and reported data updated up to June 4, 2020. (**a**) Cumulative number of cases; (**b**) cumulative number of deaths; daily (**c**) clinical, and (**d**) ICU bed requirements in the state of Bahia. The model was fitted based on data up to May 4, 2020, represented by black dots, as shown in Fig. 3. The shaded error bands represent 95% confidence intervals of the mean calculated using the weighted non-parametric bootstrap method. Gray dots depict the newly available data up to June 4, 2020. The horizontal red dashed lines in panels c and d represent, respectively, the number of beds for clinical hospitalization (466 beds) and ICUs (422 beds) available on May 4, 2020. Raw data from March 6 to June 4, 2020 are shown in this graph.

The previous analyses comprised data available up to May 4, 2020. By leveraging the most current epidemiological data available for the on-going epidemic, we were able to juxtapose the original predictions of the SEIIHURD model with the COVID-19 epidemic unfolded in Bahia up to September 13, 2020. For this ex-post assessment, we first compared 30 days predictions of the original model (calibrated on May 4) with actual data for the period (Fig. 6), reasoning that homogeneous models, such as ours, have a limited long-term prediction capacity for the state as a whole, and in a real-world situation where the model is used for predicting the allocation of healthcare resources, re-calibration with more current data would improve the accuracy of predictions while providing a reasonable time-frame for the management of resources by policy-makers.

The 30 days prediction of the original model (calibrated on May 4, 2020) was able to satisfactorily predict the number of reported cases, deaths, and ICU requirements, with real-world values falling within our predicted confidence intervals (Fig. 6). Confirming our original predictions, under a scenario where interventions were maintained, the ICU bed availability would be exhausted by May 13, while this capacity was actually reached on May 24, 2020 (11 days later) as indicated by the data, also within the range of the estimated confidence interval. A less accurate result is shown for the hospitalization requirements. With a collapse estimated to occur on May 9, the data related to clinical beds occupations only reached its capacity on May 29. It is possible that hospitalization parameters may have changed during the period, however, we were not able to obtain updated data of the hospitalization dynamics in ICM to confirm whether this actually occurred.

**Fig. 7:**
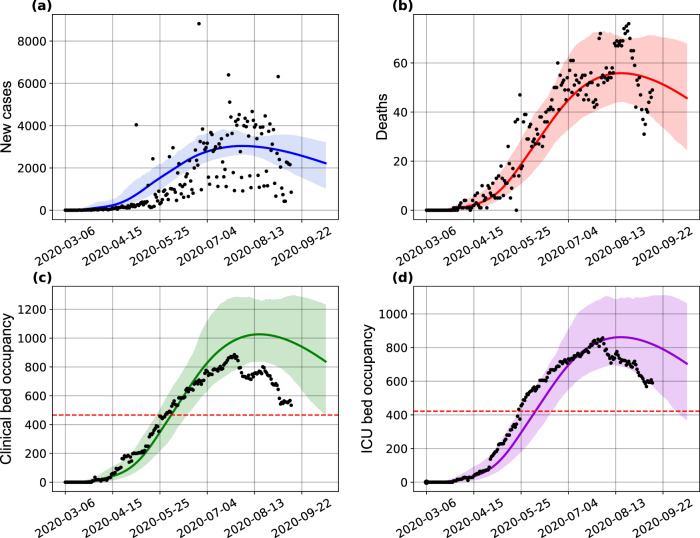
COVID-19 dynamics in Bahia. Projection of the (**a**) number of cases, (**b**) deaths, (**c**) clinical hospitalization, and (**d**) ICU bed requirements with a changing transmission rate in Bahia up to September 13, 2020. The horizontal red dashed lines in plots **c**–**d**, are, respectively, the number of beds for clinical hospitalization (466 beds) and ICUs (422 beds) available on May 4, 2020. The shaded error bands represent 95% confidence intervals of the mean calculated using the weighted non-parametric bootstrap method. The assumed parameter values are shown in Supplementary Table 4. Raw data from March 6 to September 13, 2020 are shown in this graph.

Lastly, to describe the most up-to-date transmission dynamics in Bahia, we re-estimated our model with data available up to September 13, 2020 (Fig. 7). We maintained the parameters conditions as described in “Methods”, while allowing for a new transmission rate variability. The new estimates showed a reduction of the transmission rate on June 11, hinting on an increased control of the epidemic in Bahia.

## Discussion

The COVID-19 pandemic poses unprecedented challenges to healthcare resources worldwide. Our results based on actual epidemic data and on the generalization of the SEIR model, taking into account non-detected infections, hospitalization demands, and mortality, highlight some relevant scenarios for COVID-19 in Bahia, a Brazilian state with exacerbated inequalities in health coverage and access. The trajectory of the epidemic can be characterized by the basic reproduction number (${{\mathcal{R}}}_{0}> 1$), which reflected the exponential growth of cases at the beginning of the epidemic in Salvador, the capital of Bahia, as well as its remaining 416 municipalities. We show that a reduction in disease transmission rate, as a result of non-pharmaceutical governmental interventions initiated on March 17, led to decreases in the number of cases, hospitalization demands and mortality up to May 4, which is represented in the model by a step function of transmission rate. We further show a reduction of 36% in the transmission rate in the 2 months since the first case was confirmed in Bahia. This may be partly attributed to population adherence to social distancing recommendations and convergent actions taken by local government authorities at the state and municipal levels. The effect of social distancing also became apparent in the time series modeling of the effective reproduction number, ${\mathcal{R}}(t)$, which continued to be greater than 1 (${\mathcal{R}}(t) \,> \,1$), despite the implementation of governmental measures.

Several measures to control the spread of the disease have been enforced by local governments, some of them even before the notification of the first cases of community spread, on March 19. From 17–28 March, measures were gradually applied and included the ban of public gatherings of over 50 people, closure of schools, mandatory home isolation for people with respiratory symptoms, adoption of teleworking for individuals of risk groups, and the reduction of circulation of interstate buses and intercity transportation between places where SARS-CoV-2 community transmission was declared. Concerning the latter, it is possible that the transmission rate decrease observed for the capital led to a corresponding reduction in the remaining municipalities due to decreased transportation flux of individuals.

Given the extent of measures adopted from March 17 onward, our results show that the reduction of the demand for clinical and ICU beds possibly avoided an immediate surge in hospitalization needs leading to system collapse, at least up to May 15. However, the simulated scenarios revealed that easing social distancing measures abruptly, which will result in increased transmission rates, should not be considered due to the non-linear transmission of the disease and the significant number of non-detected infections, which have been considered as the source for the majority of cases in the previous studies^10^.

Our results reinforce the negative effects on healthcare resources related to the circulation of asymptomatic/mild cases, which usually go undetected. Accordingly, policies aiming at relaxing the current level of social distancing measures, in a scenario where the majority of the population does not have access to diagnostic tests, could pose an additional burden on an already limited health system infrastructure. These results are in line with a recent study suggesting early self-isolation as a strategy to cope with the increasing demand for COVID-19-related hospitalization^18^.

Bounds for *δ* (a factor associated with the infectivity of asymptomatic/non-detected) were defined based on the previous studies^10,17,24^, which suggest that it is lower than that of symptomatic individuals (thus, *δ*  <  1). However, based on the search interval for *δ* between 0 and 0.75, simulations of a greater transmission by non-documented infections due to an increased circulation of individuals presenting with asymptomatic/mild disease can also be indicated by values of *δ* ≥ 1. The sensitivity analysis presented in our modeling results revealed the importance of increased knowledge of the extent to which infected individuals, with varying degrees of symptoms, are able to transmit the disease.

The consequences of the spread of the disease are even worse when the healthcare system is no longer able to support the number of patients needing specialized assistance–a situation referred to as a health system collapse. Our results point that non-pharmaceutical measures should be implemented in order to reduce the transmission rate of the disease, and consequently gain time to create new hospitals, acquire protective equipment material and guarantee human resources. But what can be done when faced with an already collapsed health system? We performed simulations to address this question, presenting different scenarios in order to determine an efficient strategy, by considering the period and intensity of interventions. The interventions can be applied at a single moment in time and kept until a decrease of the number of cases is observed, or a combination of interventions can be enforced at different time intervals, as proposed in other works^25,26^, depending on testing and monitoring capacities and/or local social-economical conditions. Our results show that, when faced with an already collapsed system, only vigorous measures (that reduce the transmission rate by at least 50%) enforced over at least two months or, alternatively, measures capable of reducing transmission by at least 75% over a 2-week period, are capable of re-establishing hospitalization operation capacity. Albeit harsh, other countries have successfully managed to reduce transmission at such figures by employing a myriad of public health measures (including intra-city and intercity travel restrictions, social distancing, home confinement, and centralized quarantine and expansion of available medical resources)^27^. Of note, even in the event that the transmission rate is decreased at these levels, a further, second collapse on hospitalization needs cannot be completely averted, a result which evidences the importance of timely enforcement of interventions.

By performing an ex-post assessment of the model calibrated on May 4, 2020 compared with the actual epidemic dynamics in Bahia, the usefulness of the SEIIHURD model to predict the peak demand for ICU beds in the state was illustrated, with real-world values falling within our confidence intervals. Longer-term predictions, however, are hampered by the inherent complexities of an unfolding epidemic, where human behavior, timing of deployment and lifting of governmental policies and related parameters cannot be fully expressed by modeling strategies alone. Ultimately, these will all be reflected as changes in the transmission rate over time. It should also be stressed that some of these factors may also impact directly the demand for hospitalization beds in the months following the start of the epidemic, leading to a different range of clinical hospitalizations, which may help explain the differences in the results obtained for clinical bed usage. A re-calibrated model with epidemiological data up to September 13, 2020 showed that a third decrease in the transmission rate occurred around June 11, in line with a further strengthening of state measures that took place at the end of May that could have had a positive impact on the epidemic control^28^.

Our findings have some limitations. First, this study was carried out with reported confirmed cases, which may result in an underestimation of the real incidence of COVID-19, a problem also common to other diseases^29^, as mass testing is still not performed in the country. However, the currently available national surveillance data can be considered adequate for the identification of trends of the disease, as this system is standardized and implemented in all municipalities in the country. Nevertheless, we were able to parametrize our model to a more realistic setting by using hospitalization data from a local reference infectious disease hospital currently dedicated to the care of COVID-19 patients, and the results were compared to key epidemiological parameters obtained from the literature. Thus, our modeling strategy has as an advantage being locally informed, yielding more realistic results. The implemented model does not consider the transmission by infected individuals undergoing hospitalization, although it is known that healthcare workers are more at-risk of many airborne infections, and transmission is particularly high during procedures that generate aerosols^30^. The model presented in this work is not optimal to address possible case-clustering effects, although the qualitative behavior of our results remains unaltered with respect to varying population sizes (Supplementary Note 3). Heterogeneous models can further be used to address this question, which would also require data with increased granularity. In spite of these limitations, given the general character of the mathematical model described herein, it may be readily applied to other places currently tackling the COVID-19 epidemic with simple re-estimation of the assumed values for parameters, and by taking into account the local COVID-19 epidemic situation. Notably, our predictions of the infection fatality ratio, estimated at an overall 0.69% in Bahia, are in line with current estimates drawn from multiple studies worldwide (of 0.68%)^21^, reinforcing the utility of the obtained model.

By drawing on different modeling scenarios, this work attempted to determine an efficient strategy to be employed in an effort to avoid a collapse in the local healthcare system, taking into account the length and the intensity of governmental interventions. A compromise between the availability of hospital/ICU beds and the pool of susceptible individuals was identified, for which modeling indicates the eventual occurrence of subsequent waves of infection, leading to further shortages in hospital/ICU beds. Our results underscore the crucial need for policy-makers to take into account the results of data-informed modeling when considering the lifting of restrictive measures.

## Methods

### Data sources and case definition

The models produced in this study were informed by data from multiple sources: The daily series of the cumulative confirmed COVID-19 cases and the daily mortality series for the state of Bahia, its capital Salvador and the remaining cities were obtained from publicly available data provided by the Secretary of Health of the State of Bahia (SESAB). Throughout our analyses, we consider separately the state capital (which concentrates the number of cases in the region and is an important touristic destination) and the remaining 416 municipalities. Local health authorities use the following case definition of COVID-19, based on two criteria: (i) clinical/epidemiological, namely a case of suspected flu-like syndrome or severe acute respiratory syndrome (SARS) who had contact with a laboratory-confirmed COVID-19 case in the last 7 days prior to symptoms onset; or (ii) clinical/laboratory, a suspected case of flu-like syndrome or SARS with a positive SARS-CoV-2 serology (IgM and/or IgG) or real-time PCR result.

Additionally, state-level daily hospital bed occupancy of clinical and ICU beds were provided by SESAB. By May 2020, the state of Bahia had a total of 888 hospital beds dedicated to the treatment of COVID-19 patients, of which 466 are clinical hospitalization and 422 are ICU beds. This data was not available at the municipal level; rather, due to the Brazilian administrative division of health regions, hospital bed occupancy was evaluated at the state level only. Data was available throughout the period of March 6 to May 4, 2020 for the initial analysis and up to September 13, 2020 for the ex-post assessment.

We also had access to administrative data from a reference infectious disease hospital located in Salvador (Instituto Couto Maia; ICM), in cooperation with the Rede CoVida consortium team. ICM is the leading public hospital in the state of Bahia for the treatment of COVID-19 patients. The collected hospital administrative data, aggregated across 231 patients followed from admission to discharge/death in the period of March 23 to April 16, 2020, were used to inform the hospitalization-related model parameters, including the search intervals for optimization procedures, as described in Section “Evaluation and estimation of model parameters”.

The estimated population of Bahia in 2020 was obtained from the Brazilian Institute of Geography and Statistics (IBGE).

### Assumptions and model construction

In this section, we present the SEIIHURD model that subdivides the population into eight compartments, as follows: susceptible (*S*), those who are not exposed to the disease; exposed (*E*), individuals who have been exposed to the virus and are in a latent, non-infectious period; (*I*) infectious, those currently infected and capable of transmitting the disease to contacts; recovered (*R*), those who were previously infected and recovered from the disease; deaths (*D*) those that resulted from death due to COVID-19, after passing for a period of hospitalization or ICU. The infectious individuals are further separated into asymptomatic/non-detected infections, denoted *I*_a_, and symptomatic, denoted by *I*_s_. Of note, COVID-19 transmission by undocumented infections, which encompasses truly asymptomatic (individuals that never develop symptoms) as well as those that present with very mild symptoms, has been shown to facilitate the spread of SARS-CoV-2^10^. Thus, individuals in the *I*_a_ compartment represent this group of persons that usually do not require hospitalization, are not accounted for in the official data, and define a subset of non-detected infections. However, a proportion of the infected will present with severe symptoms requiring hospitalization (clinical beds) (*H*), while those in critical conditions will eventually require ICU admission (*U*). For simplicity, we have neglected the transmission of individuals in compartments *H* and *U*, assuming that hospital containment decreases the chances of contact with susceptible individuals. We also consider a flux of patients between the *H* and *U* compartments, as individuals initially admitted to a clinical ward may worsen their condition and require an ICU bed. Furthermore, all patients in *U* are transferred to *H* prior to discharge and recovery. This assumption was based on the analysis of the administrative data from ICM, in which we observed that all patients requiring ICU beds had one of two outcomes: They were moved to hospitalization wards (H) before recovery and discharge; or they died as a result of disease. In addition, this administrative patient flow is also reported to be more common in the literature than a discharge directly home^31^. In Supplementary Fig. 6 we present the flow diagram of the proposed model. Similar works can be found in refs. ^10,14,19,32^.

To account for local interventions of movement restriction (such as governmental stay-at-home orders), we consider the transmission rate as a function of time, varying according to local measures. To define *β*, let {*t*_1_, *t*_2_, …, *t*_*n*_} be a set of points in time defining the change in the transmission rate. Then, we can write *β* as a function of time *t* as

1 $$\beta (t)={\beta }_{0}{\mathcal{H}}({t}_{1}-t) + \mathop{\sum }\limits_{i=1}^{n-1}{\beta }_{1}{\mathcal{H}}({t}_{i+1}-t){\mathcal{H}}(t-{t}_{i})+{\beta }_{0}{\mathcal{H}}(t-{t}_{n}),$$

where ${\mathcal{H}}(t)={\mathrm{lim}}_{k\to \infty }\frac{1}{1+\exp (-2kt)}$ is a Heaviside step function, *β*_*i*_ are transmission rates that can be obtained by the fitting of the data to the time interval defined by the *t*_*i*_’s. The system of differential equations then reads:

2 $$\frac{{\rm{d}}S}{{\rm{d}}t}=\frac{-\beta (t)S({I}_{s}+\delta {I}_{a})}{N},$$

3 $$\frac{{\rm{d}}E}{{\rm{d}}t}=\frac{\beta (t)S({I}_{s}+\delta {I}_{a})}{N}-\kappa E,$$

4 $$\frac{{\rm{d}}{I}_{a}}{{\rm{d}}t}=(1-p)\kappa E-{\gamma }_{a}{I}_{a},$$

5 $$\frac{{\rm{d}}{I}_{s}}{{\rm{d}}t}=p\kappa E-{\gamma }_{s}{I}_{s}, \qquad \quad $$

6 $$\frac{{\rm{d}}H}{{\rm{d}}t}=h\xi {\gamma }_{s}{I}_{s}+(1-{\mu }_{U}+{\omega }_{U}{\mu }_{U}){\gamma }_{U}U-{\gamma }_{H}H,$$

7 $$\frac{{\rm{d}}R}{{\rm{d}}t}={\gamma }_{a}{I}_{a}+(1-h){\gamma }_{s}{I}_{s}+(1-{\mu }_{H})(1-{\omega }_{H}){\gamma }_{H}H,$$

8 $$\frac{{\rm{d}}R}{{\rm{d}}t}={\gamma }_{a}{I}_{a}+(1-h){\gamma }_{s}{I}_{s}+(1-{\mu }_{H})(1-{\omega }_{H}){\gamma }_{H}H,$$

9 $$\frac{{\rm{d}}D}{{\rm{d}}t}=(1-{\omega }_{H}){\mu }_{H}{\gamma }_{H}H+(1-{\omega }_{U}){\mu }_{U}{\gamma }_{U}U,$$

where the key epidemiological parameters are described in Table 3. More details on the system of equations are provided in Supplementary Note 4.

**Table 3:** Key epidemiological parameters used in the SEIIHURD model.

Parameter	Description
*β*_0_	Pre-intervention transmission rate
*β*_1_	Post-intervention transmission rate
*t*_1_	Time of transmission rate change
*δ*	Asymptomatic/non-detected infectivity factor
*p*	Proportion of latent (E) that proceed to symptomatic infective
*κ*	Mean exposed period (days^−1^)
*γ*_a_	Mean asymptomatic period (days^−1^)
*γ*_s_	Mean symptomatic period (days^−1^)
*h*	Proportion of symptomatic needing hospitalization (clinical beds) or ICU
1 − *ξ*	Proportion of symptomatic that proceed to ICU
*γ*_*H*_	Mean hospitalization (clinical beds) period (days^−1^)
*γ*_*U*_	Mean ICU period (days^−1^)
*μ*_*H*_	Death rate of hospitalized individuals
*μ*_*U*_	Death rate of ICU individuals
*ω*_*H*_	Proportion of hospitalized that goes to ICU
*ω*_*U*_	Proportion of ICU that goes to hospitalization

### Analytical evaluation of ${{\mathcal{R}}}_{0}$ and ${\mathcal{R}}(t)$ in the SEIIHURD model

The basic reproduction number, ${{\mathcal{R}}}_{0}$, is a threshold parameter estimated in the beginning of the outbreak. It is defined by the average number of secondary infections caused by a single infective in a fully susceptible population. Under these conditions, with the model proposed here, the primary cases are generated under the initial pre-interventions transmission rate *β*(*t*) = *β*_0_^33^.

The basic reproduction number ${{\mathcal{R}}}_{0}$ has been derived within the general next generation operator framework^32,34^. It considers the unstable disease-free equilibrium point of the model, where *S* corresponds to the whole population and all other compartments are identically set to 0. Following Van den Driessche et al. (2002)^34^, the value of ${{\mathcal{R}}}_{0}$ results from a balance between the infectious and transition terms of the sub-model composed of the variables (*E*, *I*_*a*_, *I*_*s*_) associated with the transmission of the disease, which are gathered in the corresponding 3 × 3 matrices ${\mathcal{K}}$ and ${\mathcal{T}}$, given by

10 $${\mathcal{K}}=\left(\begin{array}{lll}0&{\beta }_{0}\delta &{\beta }_{0}\\ 0&0&0\\ 0&0&0\end{array}\right),$$

11 $${\mathcal{T}}=\left(\begin{array}{lll}\kappa &0&0\\ -(1-p)\kappa &{\gamma }_{a}&0\\ -p\kappa &0&{\gamma }_{s}\end{array}\right).$$

Thus, given the above matrices, ${{\mathcal{R}}}_{0}$ corresponds to the largest eigenvalue of the matrix ${\mathcal{K}}{{\mathcal{T}}}^{-1}$ and represents the sum of the contribution of the symptomatic and asymptomatic/non-detected transmission, being expressed by

12 $${{\mathcal{R}}}_{0}=\frac{{\beta }_{0}p}{{\gamma }_{s}}+\frac{{\beta }_{0}\delta (1-p)}{{\gamma }_{a}}.$$

The effective reproduction number ${\mathcal{R}}(t)$ provides a measure of how the newly infected part of the population will further transmit the pathogen as the epidemic evolves over time. Indeed, as reminded above, the evaluation of ${{\mathcal{R}}}_{0}$ considers that the whole population is initially susceptible, a condition that is strictly valid only when the pathogen is first introduced into the system. As time evolves, the susceptible fraction of the population always decreases with time for models where the *R* compartment does not feed *S*. It is still unknown whether re-infection by SARS-CoV-2 can occur, but initial evidence suggests against this possibility^35^. Here we assume that only a single COVID-19 infection event can occur in any single person.

The epidemiological meaning of ${\mathcal{R}}(t)$ is the same as for ${{\mathcal{R}}}_{0}$, namely, it represents the average number of secondary infections that an individual, who became infected at time *t*, is able to generate. The series of ${\mathcal{R}}(t)$ values indicates the current trend of the epidemic and captures changes caused by recently-introduced interventions (such as governmental policies) or by natural decrease of the susceptible population. Accordingly, it provides a quantitative evidence of whether further measures are needed to control the epidemic. Here ${\mathcal{R}}(t)$ has been estimated following the assumptions introduced in Wallinga, Jacco, and Marc Lipsitch (2007)^36^. It is based on the series of daily infected individuals, that is considered here as the source to insert into the general renewal equation for a birth process

13 $${\mathcal{R}}(t)=\frac{J(t)}{\mathop{\int}\nolimits_{0}^{\infty }J(t-x)g(x)dx},$$

with *J*(*t*) = *b*(*t* + *ℓ*) where *b*(*t*) is the number of daily reported cases.

In an epidemiological context, *J*(*t*) represents the daily number of new infections, while *g*(*x*) is the disease probability distribution function for the time an individual takes to infect secondary cases, in accordance with the ideas in the original infection-age model by Kermack and McKendrick^9^. In this case, the function *g*(*x*) receives contributions that depend on the dynamics of the same three compartments *E*, *I*_*a*_, *I*_*s*_ that impact the evaluation of ${{\mathcal{R}}}_{0}$. The population flow through these compartments consists of a first sequential step (*E*) followed by a bifurcation event leading either to the asymptomatic/non-detected (*I*_*a*_) or the symptomatic (*I*_*s*_) compartments. Therefore, it is first necessary to separately evaluate the composite functions *g*_*a*_(*x*) and *g*_*s*_(*x*) that account, respectively, for the sequential contributions of the flows *E* → *I*_*a*_ → *O* and *E* → *I*_*s*_ → *O*, where *O* indicates any compartment not responsible for infectious steps. Here we follow the framework developed by Brauer^37^ to obtain the following expressions:

14 $${g}_{a}(t)=\frac{\kappa {\gamma }_{a}}{{\gamma }_{a}-\kappa }({{\rm{e}}}^{-\kappa t}-{{\rm{e}}}^{-{\gamma }_{a}t}),\qquad \qquad \qquad {g}_{s}(t)=\frac{\kappa {\gamma }_{s}}{{\gamma }_{s}-\kappa }({{\rm{e}}}^{-\kappa t}-{{\rm{e}}}^{-{\gamma }_{s}t}).$$

Subsequently, these two contributions are combined, leading to the expression:

15 $$g(x)=\frac{{g}_{s}(x)p\beta /{\gamma }_{s}+{g}_{a}(x)(1-p)\beta \delta /{\gamma }_{a}}{p\beta /{\gamma }_{s}+(1-p)\beta \delta /{\gamma }_{a}}.$$

The details of the evaluation of Eqs. (14) and (15) are available in Supplementary Note 5, where we also detail how the expressions in Equation (14) should be replaced when *κ* = *γ*_*a*_ or *κ* = *γ*_*s*_. It is noteworthy to see that the weights in factors multiplying *g*_*a*_(*x*) and *g*_*s*_(*x*) correspond to the contributions of the corresponding flow paths to ${{\mathcal{R}}}_{0}$ in Eq. (12), with the difference that the value of *β*_0_ is replaced by the value of *β* at the current time *t*.

In order to overcome the fluctuations of the confirmed number of cases (which is influenced by testing capacity and its associated increase, even if momentarily, such as when pending tests accumulate), we present two series of ${{\mathcal{R}}}_{t}$, one calculated from the daily number of confirmed cases, as informed by local health authorities; and another ${{\mathcal{R}}}_{t}$ series where this data is informed by the predictions of the model. More details are given in Supplementary Note 5.

### Parameter sensitivity analysis

Sensitivity analysis was conducted to assess the effects of model parameters in the dynamics of *I*_*a*_, *I*_*s*_, *U*, *H,* and *D* over time. By using an statistical variance-based method, described by Sobol (2001)^38^, the sensitivity analysis of the system described by Eqs. (2)–(9) considers the following parameter vector

16 $$\theta := \left({\beta }_{0},{\beta }_{1},{\gamma }_{H},{\gamma }_{U},\delta ,h,{t}_{1},k\right)\in {{\mathbb{R}}}^{8},$$

and assumes that its elements are uniformly distributed in proper intervals as follows:

17 $${\beta }_{0} 	\sim {\mathcal{U}}(0,2),\qquad {\beta }_{1} \sim {\mathcal{U}}(0,2),\quad\qquad {\gamma }_{H} \sim {\mathcal{U}}(1/12,1/4),\quad {\gamma }_{U} \sim {\mathcal{U}}(1/12,1/3),\\ \delta 	\sim {\mathcal{U}}(0,0.75),\quad h \sim {\mathcal{U}}(0.05,0.25),\quad {t}_{1} \sim {\mathcal{U}}(0,30),\quad \qquad k \sim {\mathcal{U}}(0,100).$$

This method is divided into two steps, described in more detail in Supplementary Note 2. The numerical simulations were performed using the SALib library^39^, and the experiments were conducted generating *N* = 12,000 parameter combinations, totaling 120,000 simulations of the model. The influence of each parameter on the model dynamics was evaluated using the total effect index, which takes into account higher-order interactions amongst the variables of the model.

### Evaluation and estimation of model parameters

The estimation of the parameters occurred within a chosen range based on literature and data collected locally, as described in Supplementary Tables 1 and 2. The initial conditions (*S*_0_, *I*_*a*,0_, *I*_*a*,0_, *E*_0_, *R*_0_, *H*_0_, *U*_0_, *D*_0_) is given by (1 − *I*_*a*,0_ − *I*_*s*,0_ − *E*_0_, *I*_*s*,0_, *I*_*a*,0_, *I*_*s*,0_, *E*_0_, 0, 0, 0, 0).

The parameters *p*, *κ*, *γ*_*a*_, *γ*_*s*_, *ξ*, *ω*_*U*_, *ω*_*H*_, *μ*_*U*_, *μ*_*H*_ were kept fixed and the remaining were obtained by estimating the best values that fit the model to the data. To define the fixed parameters and the search intervals to use for the estimations, we performed a literature review of published papers and collected the data regarding key epidemiological parameters that inform our model (see Supplementary Table 1).

As an additional guide to obtain a locally-informed model, administrative data from a reference infectious disease hospital (ICM; see Data Sources section) were used in order to capture plausible ranges for the hospitalization-related model parameters: Mean hospitalization period (*γ*_*H*_), mean ICU period (*γ*_*U*_), death rate of hospitalized individuals (*μ*_*H*_), death rate of ICU individuals (*μ*_*U*_), proportion of clinically hospitalized transferred to ICU (*ω*_*H*_) and proportion of ICU individuals that return to clinical hospitalization (*ω*_*u*_) (see Supplementary Table 2).

Based on the search ranges, optimized model parameters were estimated using the Particle Swarm Optimization (PSO) metaheuristic^40^. Under the PSO framework, we used a multi-optimization function to simultaneously optimize the model to the series of daily confirmed cases, deaths, clinical hospitalization, and ICU occupations in the whole state, and to daily confirmed cases and deaths for the capital city Salvador and the remaining 416 municipalities (given that the hospitalization series were only available aggregated at the statewide level) up to May 4, 2020. PSO was implemented using pyswarms library version 1.1.0 for Python 3^41^, and was executed with 300 particles through 1,000 iterations with cognitive parameter 0.1, social parameter 0.3, inertia parameter 0.9, evaluating five closest neighbors through Euclidean (or L2) distance metric. In addition to the point estimates obtained by the PSO method for the parameters *β*_0_, *β*_1_, *δ*, *h*, *γ*_*H*_ and *γ*_*U*_, percentile confidence intervals were also estimated for these parameters. The intervals were constructed using the weighted non-parametric bootstrap method^42^, considering 500 replicates of the original series *S*_*t*_, *t* = 1, …, *n*, which represents the number of new cases observed at time *t*. The proportion of observed cases at time *t* (number of cases at time *t*/total number of cases in the analyzed period) was used as a weight in the re-sampling process to obtain the bootstrap replicates. Then, the SEIIHURD model was adjusted for each replicated series and the estimates obtained for the model parameters were stored in vectors, generating the empirical distribution for each parameter.

Given the complexity of the proposed model, we performed an identifiability analysis in terms of the number of parameters and possible correlations between them. A simulation study was carried out in order to assess the identifiability of the model presented. The study was conducted based on the approach developed by Roosa and Chowell^43^, which makes use of the parametric bootstrap method to generate data from a system of dynamic equations, in order to quantify the uncertainty and assess the identifiability of the indicators of the model. More information is given in Supplementary Note 6.

### Modeling scenarios

We present our analysis as follows: First, we study the effects of previously enforced interventions in the state of Bahia, its capital city Salvador and the remaining 416 municipalities. For this, we considered the SEIIHURD model with the proportion of symptomatic needing hospitalization or ICU (parameter *h*) equals to zero, so that the resulting model does not consider the compartments of hospitalization, ICU, and death, effectively resembling an SEIR model with asymptomatic/non-detected transmission.

Then, we analyze these effects on the number of deaths and hospitalization requirements in the state level. We show the impact of the non-detected individuals on the dynamics of COVID-19 transmission.

Lastly, to study the future behavior of the transmission of the disease in Bahia, we simulated different scenarios that may impact the number of cases, mortality, and healthcare demands. The following scenarios were considered: (1) An immediate intervention taking place on May 5, sustained for a period of 7, 14, or 30 days, and resulting in a reduction of the transmission rate by 25%, 50%, or 75%; (2) A critical intervention, adopted when the collapse of clinical bed occupancy occurs (on May 14, 2020, the predicted date of peak demand), maintained for a period of 7, 14, 30, 60, 90 days and leading to a reduction of the transmission rate by 25%, 50% or 75%; and (3) implementations of more than two interventions in sequence.

### Ethics statement

This study was conducted with publicly available data from the COVID-19 epidemic in Bahia, obtainable from the periodic epidemiological bulletins published by the Secretary of Health of the State of Bahia (SESAB), as well as with aggregated administrative data from Instituto Couto Maia hospital (Salvador, Bahia), and therefore no approval by an ethics committee was required, according to Resolutions 466/2012 and 510/2016 (article 1, sections III, and V) from the National Health Council (CNS), Brazil.

## Reporting summary

Further information on research design is available in the Nature Research Reporting Summary linked to this article.

## Supplementary information

Supplementary Information

Reporting Summary

## Data Availability

The series of COVID-19 cases and deaths and hospital occupancy are publicly available from the State Secretary of Health of Bahia (SESAB) at https://infovis.sei.ba.gov.br/covid19/ and are also available as CSV files. All other data, including the list of parameters used to inform the model, are presented within the Supplementary Material and in the GitHub repository at https://github.com/cidacslab/Mathematical-and-Statistical-Modeling-of-COVID19-in-Brazil.git^44^. Raw data is included within Figs. 1, 3, 4, 5, 6, and 7. Source data are provided with this paper.

## Source data

Source Data

## Code availability

Codes used to produce the results presented herein are available in a public GitHub repository at https://github.com/cidacslab/Mathematical-and-Statistical-Modeling-of-COVID19-in-Brazil.git^44^, as well as in the Supplementary Material.
