# Supplementary material for: Mathematical modeling of COVID-19 in 14.8 million individuals in Bahia, Brazil
Source: Nat Commun. 2021 Jan 12;12:333. doi: 10.1038/s41467-020-19798-3 (PMC7803757; doi:10.1038/s41467-020-19798-3)
Supplement: Supplementary file 1 — Supplementary Information [file 41467_2020_19798_MOESM1_ESM.pdf]

Mathematical modelling of COVID-19 in 14.8 million individuals in Bahia, Brazil

Juliane F. Oliveira[1,2,*], **Daniel C. P. Jorge**[3], **Rafael V. Veiga**[1], **Moreno S. Rodrigues**[4], **Matheus F. Torquato**[5], **Nívea B. da Silva**[6], **Rosemeire L. Fiaccone**[6], **Luciana L. Cardim**[1], **Felipe A. C. Pereira**[7], **Caio P. Castro**[3], **Aureliano S. S. Paiva**[1], **Alan A. S. Amad**[5], **Ernesto A. B. F. Lima**[8], **Diego S. Souza**[1], **Suani T. R. Pinho**[3,**], **Pablo Ivan P. Ramos**[1,**], **Roberto F. S. Andrade**[1,3,**]

[1]Center of Data and Knowledge Integration for Health (CIDACS), Instituto Gonçalo Moniz, Fundação Oswaldo Cruz, Salvador, Bahia, Brazil
[2]Centre of Mathematics of the University of Porto (CMUP), Department of Mathematics, Porto, Portugal
[3]Instituto de Física, Universidade Federal da Bahia, Salvador, Bahia, Brazil
[4]Fundação Oswaldo Cruz, Porto Velho, Rondônia, Brazil
[5]College of Engineering, Swansea University, Swansea, Wales, United Kingdom
[6]Instituto de Matemática e Estatística, Universidade Federal da Bahia, Salvador, Bahia, Brazil
[7]Instituto de Física, Universidade de São Paulo, São Paulo, Brazil
[8]Oden Institute for Computational Engineering and Sciences, The University of Texas at Austin, Austin, Texas, United States of America
[*]Correspondence: Juliane F. Oliveira (julianlanzin@gmail.com).
[**]These authors contributed equally to this work.

ABSTRACT

In this document we present the relevant Supplementary Information accompanying the manuscript "Mathematical modelling of COVID-19 in 14.8 million individuals in Bahia, Brazil" by Oliveira *et al*.

Supplementary Figures

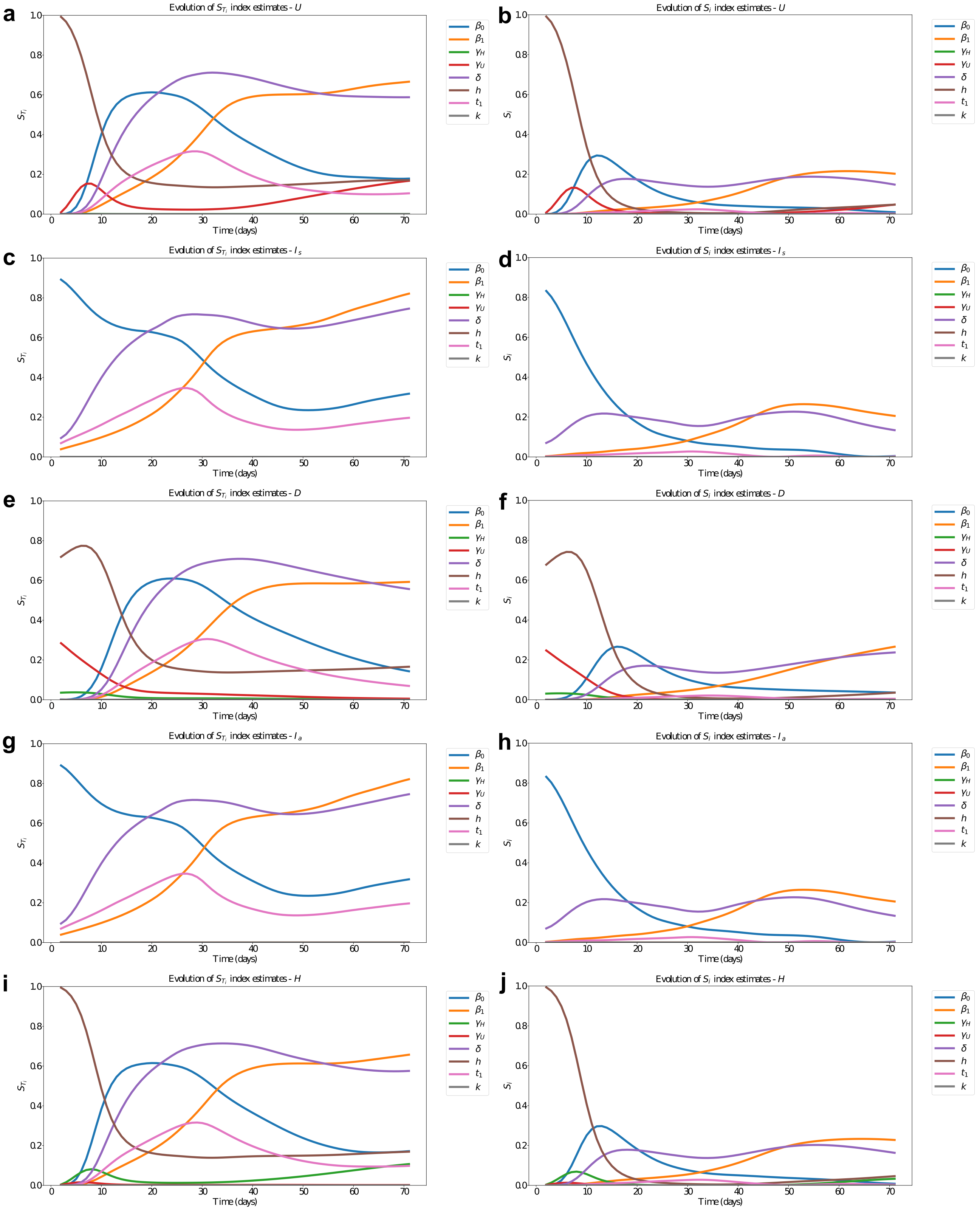

Supplementary Figure 1. Sensitivity analysis study for I_a, I_s, U, H and D compartments over time. The analysis of the total effect index, S_T, shown in the left-hand graphs (subplots a,c,e,g,i), for the 8 parameters evaluated, indicates that δ, β_0, β_1, and h are the most influential parameters to the model output. Also, the mean exposed period, k, is the less influential parameter, indicated by the low S_T. The right-hand graphs (subplots b,d,f,h,j) show the first-order effects, which do not take into account higher-order interactions among variables, evidencing the non-linearity that characterizes the SEIIHURD model. The parameter search intervals follow those presented in Eq. 2.

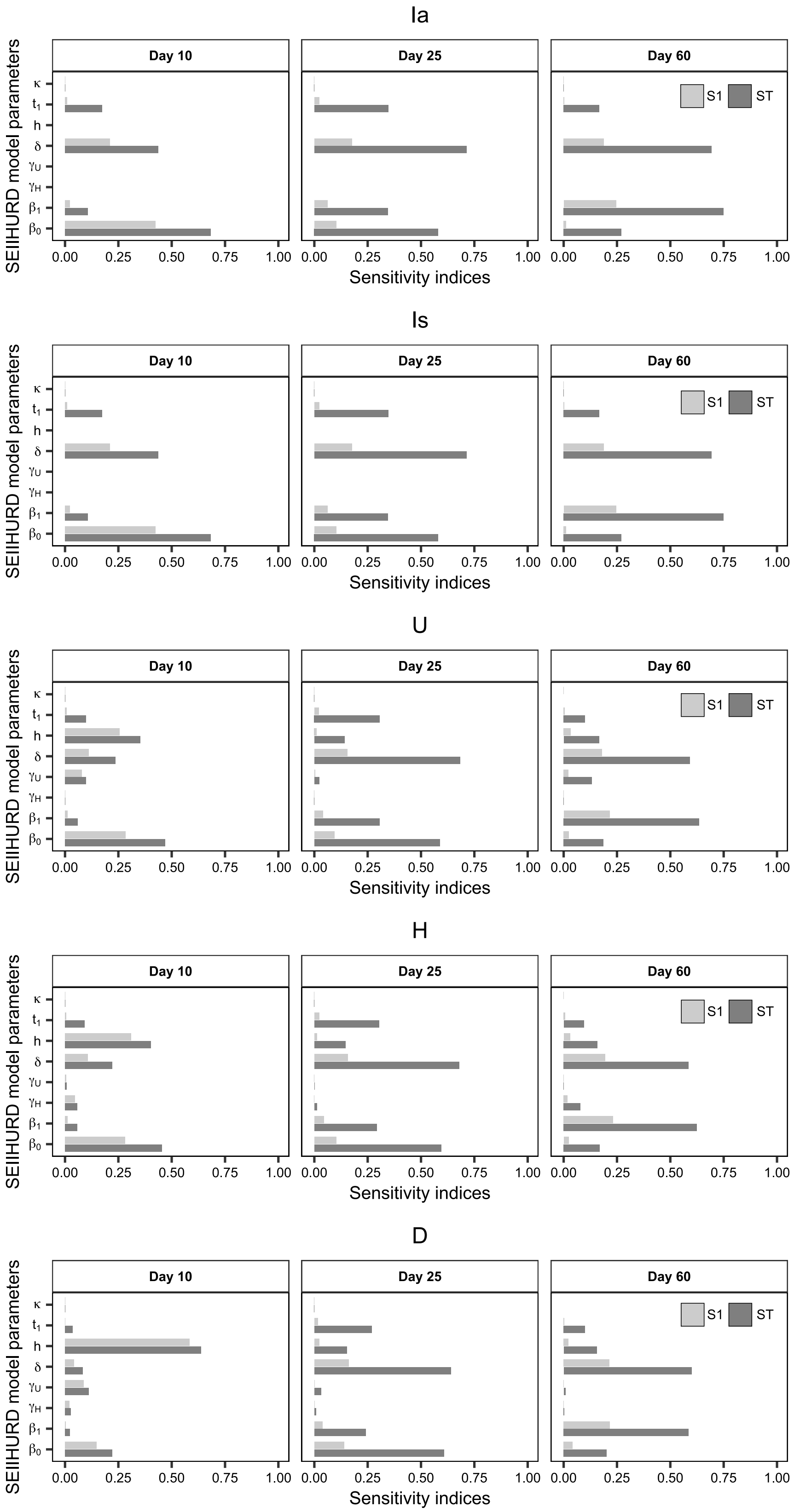

Supplementary Figure 2. A snapshot of the sensitivity analysis study for I_a, I_s, U, H and D compartments in selected simulation periods. The total effect index (S_T) and the first-order effects (S_1) are shown for each variable. Three simulation periods were chosen for this analysis (10 days, 25 days and 60 days). The parameter search intervals follow those presented in Eq. 2.

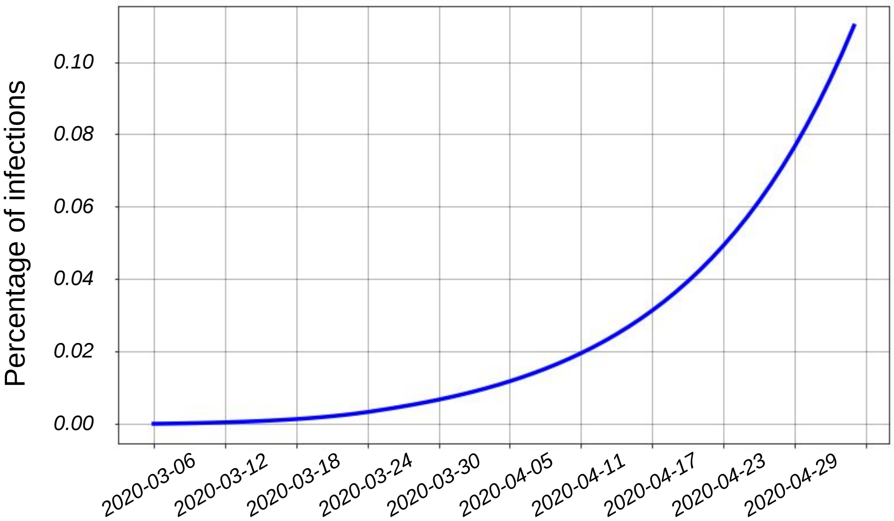

Supplementary Figure 3. Percentage of infections over time. Percentage of the population infected by SARS-CoV-2 over time inferred by the SEIIHURD model applied to Bahia up to May 4, 2020. The plot accounts for both symptomatic and asymptomatic/mild infections.

(a)

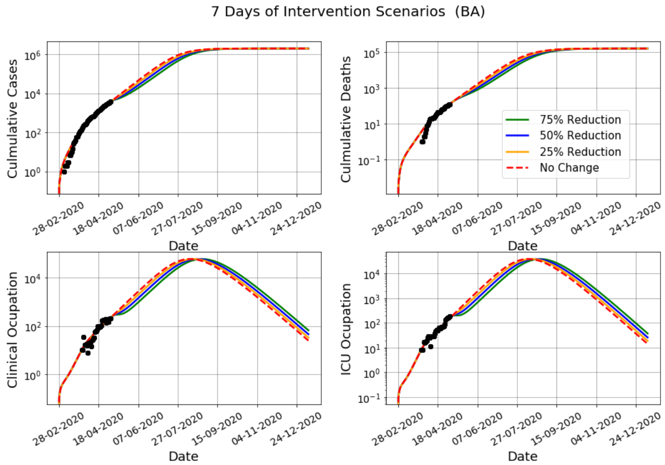

(b)

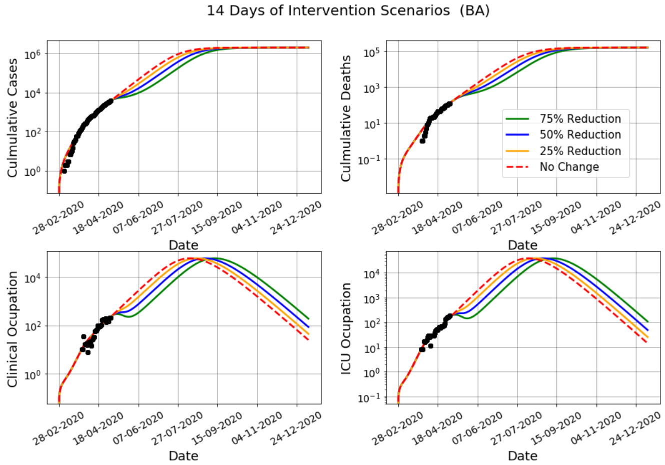

(c)

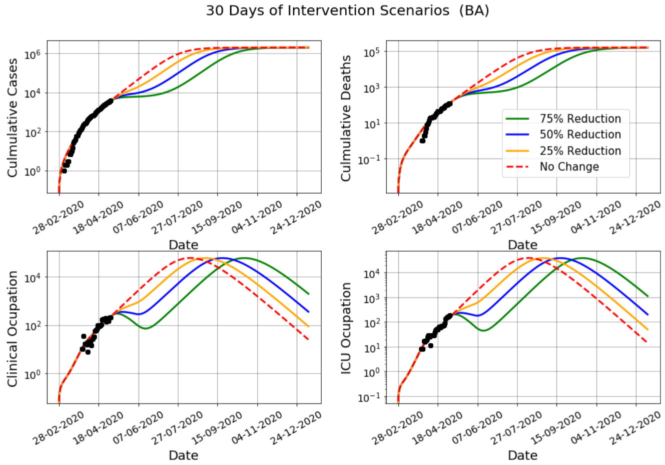

Supplementary Figure 4. Effect of the enforced interventions on the number of deaths, clinical hospitalizations and ICU requirements in Bahia after immediate intervention on May 2. Scenarios of 25%, 50% and 75% reduction of the transmission rate β_1 represented by the different colours in each panel. The effects of the duration of measures maintained by 7, 14, and 30 days are shown in panels a–c, respectively. The red dashed line represents the scenario where no change in the transmission rate is observed (ie. absence of interventions).

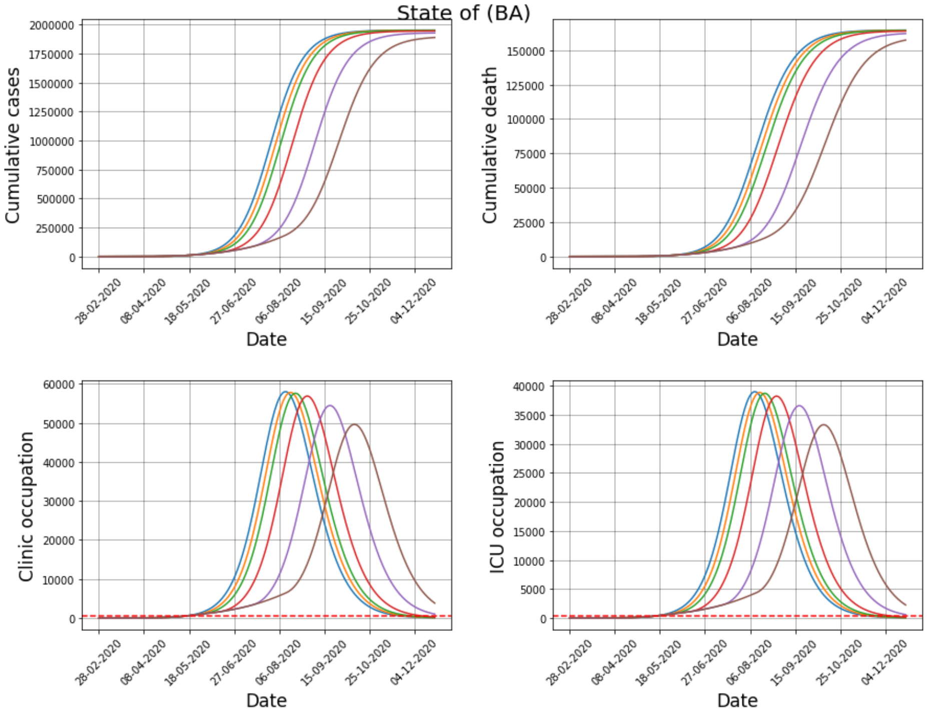

Supplementary Figure 5. Effect of the enforced interventions on the number of deaths, clinical hospitalizations and ICU requirements in Bahia on the collapse of healthcare system. The simulations are presented with a reduction of 25% of the transmission rate β_1. The different colours represent the duration of the measures: 7 (orange), 14 (green), 30 (red), 60 (violet) and 90 (brown) days. The horizontal red dashed lines are, respectively, the current capacity for beds for clinical hospitalization (466 beds) and ICUs (422 beds). The blue line represents the current scenario.

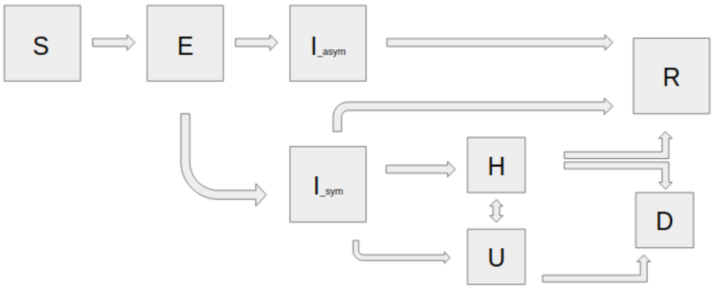

Supplementary Figure 6. Compartmental modelling. Flow diagram for modelling the dynamics of COVID-19 transmission in the 8-compartment SEIIHURD model.

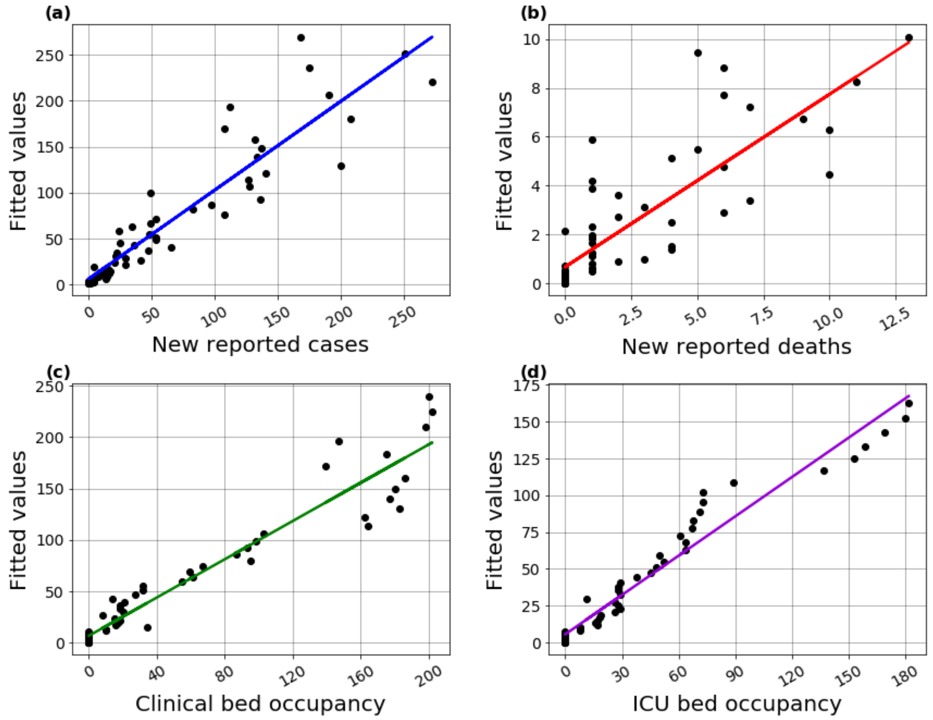

Supplementary Figure 7. Residuals analysis. Plot of the residuals between the data and simulations of (a) cases, (b) deaths, (c) clinical hospitalizations and (d) ICU requirements in Bahia up to May 4, 2020.

Supplementary Tables

Supplementary Table 1. Literature review of key epidemiological parameters used in the SEIIHURD model, with their mean estimates (or range[s]) obtained from the referenced works.

Parameter	Interpretation	Type	Value or range	Reference
N	Size of initial population.	constant	14,873,064 for the state of Bahia; 2,831,557 for Salvador; and 12,098,867 for inland cities.	[4]
β	Transmission rate that varies over time.	stepwise function	[0.5944, 1.68]	[5]
		constant (with median and 95% CI)	1.12 (1.07, 1.17)	[6][a]
		constant (with median and 95% CI)	0.51 (0.37, 0.68)	[6][b]
		constant (with median and 95% CI)	0.35 (0.30, 0.52)	[6][c]
δ	Asymptomatic/non-detected infectivity factor.	constant (with median and 95% CI)	0.55 (0.49, 0.60)	[6][a]
		constant (with median and 95% CI)	0.47 (0.36, 0.64)	[6][b]
		constant (with median and 95% CI)	0.42 (0.34, 0.61)	[6][c]
		constant	0.1	[7]
		constant	0.5	[8]
κ	Mean exposed period, or incubation time (days^{-1}).	constant	3	[5,9]
		constant (with median and 95% CI)	3.68 (3.48, 3.90)	[6][a]
		constant (with median and 95% CI)	3.62 (3.44, 3.87)	[6][b]
		constant (with median and 95% CI)	3.43 (3.30, 3.63)	[6][c]
		constant (with mean and 95% CI)	5.2 (4.1, 7.0)[d]	[10]
		constant (with mean and 95% CI)	6.4 (5.7-7.7) (range: 2.1-11.1)	[11]
		constant (with mean and 95% CI)	5.6 (5.0-6.3)[e]	[12]
		constant (with mean and 95% CI)	4.2 (3.5-5.1)	[13]
		constant	4.6	[9]
		constant	5.1	[8]
		constant (with mean and 95% CI)	5.1 (4.5-5.8)	[14]
		median (and IQR)	4 (2-7)	[15]
p	Proportion of latent (E) that proceed to symptomatic infective.	constant	0.14	[6][f]
		constant	0.6 [4]	[7]
		constant (with mean and 95% CI)	0.821 (0.798-0.845)	[16][g]
		constant (with mean and 95% CI)	0.692 (0.462-0.923)	[17]
γ_a	Mean asymptomatic period (days^{-1}).	constant (with median and 95% CI)	3.45 (3.24, 3.70)	[6][a]
γ_s	Mean symptomatic period (days^{-1}).	constant	5	[5,9]
		constant (with median and 95% CI)	3.47 (3.26, 3.67)	[6][a]
		constant (with median and 95% CI)	3.15 (2.62, 3.71)	[6][b]
		constant (with median and 95% CI)	3.32 (2.92, 4.04)	[6]
h	Proportion of symptomatic needing hospitalization or ICU (severe or critical cases).	constant	0.2	[5][h]
		constant	0.044	[9]
		constant	0.05	[18]
$1-\xi$	Proportion of hospitalized symptomatic that proceed to ICU (critical cases).	constant	0.0132	[9]
		constant	0.3	[8]
		constant	0.325	[19]
		constant	0.05	[15]
		constant	0.26	[20,21]
		constant	0.088	[22]
γ_H	Mean hospitalization (clinical beds) period (days^{-1}).	constant	8	[8,9]
		median (and IQR)	4 (2-6)	[23]
γ_U	Mean period in ICU (days^{-1}).	constant	10	[8,9]
		median (and IQR)	6 (3-9)	[23]
		median (and IQR)	8 (4-12)	[19]
μ_H	Death rate of individuals in general ward.	constant	0.185	[23]
μ_U	Death rate of individuals in ICU.	constant	0.5	[8]
		constant	0.45	[23]
		constant	0.49	[19]
ω	proportion of patients in general ward transferred to ICU (critical cases).	constant	0.281	[23]

[a] metapopulation model adjusted to include separate mean infectious periods for documented and undocumented infections.
[b] model with no travel between cities (Jan 24-Feb 3).
[c] model with no travel between cities (Jan 24-Feb 8).
[d] among 10 confirmed cases.
[e] n = 158 including Wuhan residents.
[f] in this study, the term undocumented infection is loosely used to include those infected with only mild, limited or no symptoms.
[g] sensitivity analysis considering a range in the incubation period yielded estimates (and 95% CI) of the asymptomatic proportion ranging from 20.6% (18.5-22.8%) to 39.9% (35.7-44.1%).
[h] no distinction between general beds or ICU requirements were made.

Supplementary Table 2. Epidemiological parameters associated to hospitalization dynamics of patients with COVID-19 from the Couto Maia Institute (ICM)*.

Parameter	Parameter search interval (ICM)	PSO estimate/fixed
$1-\xi$	0.50	0.47
γ_H	[1/20, 1]	0.13
γ_U	[1/20, 1]	0.14
μ_H	0.11	0.15
μ_U	0.23	0.40
ω_H	0.14	0.14
ω_U	0.29	0.29

$*$ The mean period in clinical hospitalization, γ_H, is 4.3 days
ICU period, γ_H, is 4.7 days.

Supplementary Table 3. Key epidemiological parameters used in the SEIIHURD model, with their search interval and respective estimates obtained according to best fit for Salvador, inland cities and the whole state, with data up to May 4th. The parameters search intervals were informed by values in Tables 1 and 2.

Parameter	Description	Interval	Fixed	Estimated (95% CI) Bahia	Estimated (95% CI) Salvador	Estimated (95% CI) Other†
β_0	Pre-intervention transmission rate	$[0,2]$	-	1.28 (1.26 - 1.30)	1.28 (1.27 - 1.30)	0.97 (0.95 - 0.99)
β_1	Post-intervention transmission rate	$[0,2]$	-	0.92 (0.90 - 0.93)	0.58 (0.57 - 0.59)	0.57 (0.56 - 0.58)
t_1	Time of transmission rate change	[March 15th, April 15th]		April 2nd	March 26th	April 03rd
δ	Asymptomatic/non-detected infectivity factor	$[0,0.75]$	-	0.34 (0.33 - 0.35)	0.70 (0.69 - 0.72)	0.62 (0.60 - 0.64)
p	Proportion of latent (E) that proceed to symptomatic infective	$[0.13,0.5]$	0.2	-	-	-
κ	Mean exposed period	$[1/6,1/3]$	1/4	-	-	-
γ_a	Mean asymptomatic period	$[1/3.70,1/3.24]$	1/3.5	-	-	-
γ_s	Mean symptomatic period	$[1/5,1/3]$	1/4	-	-	-
h	Proportion of symptomatic needing hospitalization or ICU	$[0.05,0.25]$	-	0.28 (0.28, 0.29)	-	-
$1-\xi$	Proportion of symptomatic that proceed to ICU	$[0.01,0.5]$	0.47	-	-	-
γ_H	Mean hospitalization (clinical beds) period	$[1/12,1/4]$	-	0.13 (0.13, 0.14)	-	-
γ_U	Mean ICU period	$[1/12,1/3]$	-	0.14 (0.13, 0.14)	-	-
μ_H	Death rate of hospitalized individuals	$[0.1,0.2]$	0.15	-	-	-
μ_U	Death rate of ICU individuals	$[0.4,0.5]$	0.4	-	-	-
ω_H	Proportion of hospitalized that goes to ICU	$[0.1,0.3]$	0.14	-	-	-
ω_U	Proportion of ICU that goes to hospitalization	$[0.1,0.3]$	0.29	-	-	-

†, Other refers to the remaining 416 state municipalities.

Supplementary Table 4. Key epidemiological parameters used in the SEIIHURD model, with their search interval and respective estimates obtained according to best fit to data up to September 13, 2020 for Bahia. The parameters search intervals were informed by values in Tables 1 and 2.

Parameter	Description	Interval	Fixed	Estimated (95% CI)
β_0	Pre-intervention transmission rate	$[0,2]$	-	1.40 (1.37 - 1.43)
β_1	Post-intervention transmission rate	$[0,2]$	-	0.96 (0.94 - 0.98)
β_2	Post-intervention transmission rate	$[0,2]$	-	0.66 (0.65 - 0.68)
t_1	Time of transmission rate change	[March 15th, April 15th]	-	April 3nd
t_2	Time of transmission rate change	[April 15th, September 13th]	-	June 11th
δ	Asymptomatic/non-detected infectivity factor	[0,0.75]	-	0.31 (0.30 - 0.32)
p	Proportion of latent (E) that proceed to symptomatic infective	$[0.13,0.5]$	0.2	-
κ	Mean exposed period	$[1/6,1/3]$	$1/4$	-
γ_a	Mean asymptomatic period	$[1/3.70,1/3.24]$	$1/3.5$	-
γ_s	Mean symptomatic period	$[1/5,1/3]$	$1/4$	-
h	Proportion of symptomatic needing hospitalization or ICU	$[0.05,0.25]$	-	0.06 (0.06, 0.064)
$1-\xi$	Proportion of symptomatic that proceed to ICU	$[0.01,0.5]$	0.47	-
γ_H	Mean hospitalization (clinical beds) period	$[1/12,1/4]$	-	0.18 (0.17 - 0.18)
γ_U	Mean ICU period	$[1/12,1/3]$	-	0.13 (0.13, 0.14)
μ_H	Death rate of hospitalized individuals	$[0.1,0.2]$	0.15	-
μ_U	Death rate of ICU individuals	$[0.4,0.5]$	0.4	-
ω_H	Proportion of hospitalized that goes to ICU	$[0.1,0.3]$	0.14	-
ω_U	Proportion of ICU that goes to hospitalization	$[0.1,0.3]$	0.29	-

Supplementary Note 1

Additional list of members of the *Rede CoVida Modelling Task-force*

Rede CoVida Modelling Task-force

All authors listed in the author list in the main manuscript are members of the *Rede CoVida Modelling Task-force* consortium. We list bellow the additional members that we would like to acknowledge, in alphabetical ordering:

- Ceuci Nunes
  - Instituto Couto Maia, Salvador, Bahia, Brazil
- Gervásio F. Santos
  - Faculdade de Economia, Universidade Federal da Bahia, Salvador, Bahia, Brazil
- José G. B. Castro
  - Instituto de Física, Universidade Federal da Bahia, Salvador, Bahia, Brazil
- José G. V. Miranda
  - Instituto de Física, Universidade Federal da Bahia, Salvador, Bahia, Brazil
- Luis Eugênio P. F. de Souza
  - Instituto de Saúde Coletiva, Universidade Federal da Bahia, Salvador, Bahia, Brazil
- Maira L. Souza
  - Center of Data and Knowledge Integration for Health (CIDACS), Instituto Gonçalo Moniz, Fundação Oswaldo Cruz, Bahia, Brazil
- Maria Yury Ichihara
  - Center of Data and Knowledge Integration for Health (CIDACS), Instituto Gonçalo Moniz, Fundação Oswaldo Cruz, Bahia, Brazil
- Mateus S. Silva
  - Instituto de Física, Universidade Federal da Bahia, Salvador, Bahia, Brazil
- Maurício L. Barreto
  - Center of Data and Knowledge Integration for Health (CIDACS), Instituto Gonçalo Moniz, Fundação Oswaldo Cruz, Bahia, Brazil
  - Instituto de Saúde Coletiva, Universidade Federal da Bahia, Salvador, Bahia, Brazil
- Raphael S. do Rosário
  - Instituto de Física, Universidade Federal da Bahia, Salvador, Bahia, Brazil
- Vivian A. F. Silva
  - Center of Data and Knowledge Integration for Health (CIDACS), Instituto Gonçalo Moniz, Fundação Oswaldo Cruz, Bahia, Brazil

Supplementary Note 2
Parameter sensitivity analysis

Parameter sensitivity analysis

Sensitivity analysis was conducted to assess the effects of model parameters in the dynamics of I_a, I_s, U, H and D over time. By using an statistical variance-based method, described by Sobol (2001)[1], the sensitivity analysis of the system described by Eqs.(2)-(9) in the main text, considers the following parameter vector

$$\theta := (\beta_0, \beta_1, \gamma_H, \gamma_U, \delta, h, t_1, k) \in \mathbb{R}^8, \tag{1}$$

and assumes that its elements are uniformly distributed in proper intervals as follows:

$$\begin{array}{llll} \beta_0 \sim \mathscr{U}(0,2), & \beta_1 \sim \mathscr{U}(0,2), & \gamma_H \sim \mathscr{U}(1/12,1/4), & \gamma_U \sim \mathscr{U}(1/12,1/3), \\ \delta \sim \mathscr{U}(0,0.75), & h \sim \mathscr{U}(0.05,0.25), & t_1 \sim \mathscr{U}(0,30), & k \sim \mathscr{U}(0,100). \end{array} \tag{2}$$

This method is divided in two steps. The first one consists in generating the sample values for the input factors in Eq. (1) by creating matrices A and B, each one with size $N \times n$, where N is the number of samples and $n = 8$ is the number of parameters being analyzed, given by

$$A = \begin{pmatrix} \theta_1^{(A1)} & \theta_2^{(A1)} & \dots & \theta_i^{(A1)} & \dots & \theta_n^{(A1)} \\ \theta_1^{(A2)} & \theta_2^{(A2)} & \dots & \theta_i^{(A2)} & \dots & \theta_n^{(A2)} \\ \vdots & \vdots & \dots & \vdots & \dots & \vdots \\ \theta_1^{(AN)} & \theta_2^{(AN)} & \dots & \theta_i^{(AN)} & \dots & \theta_n^{(AN)} \end{pmatrix} \tag{3}$$

and

$$B = \begin{pmatrix} \theta_1^{(B1)} & \theta_2^{(B1)} & \dots & \theta_i^{(B1)} & \dots & \theta_n^{(B1)} \\ \theta_1^{(B2)} & \theta_2^{(B2)} & \dots & \theta_i^{(B2)} & \dots & \theta_n^{(B2)} \\ \vdots & \vdots & \dots & \vdots & \dots & \vdots \\ \theta_1^{(BN)} & \theta_2^{(BN)} & \dots & \theta_i^{(BN)} & \dots & \theta_n^{(BN)} \end{pmatrix}. \tag{4}$$

Then, n matrices A_B^i are created, where column i comes from matrix B and all other $n-1$ columns come from matrix A

$$A_B^i = \begin{pmatrix} \theta_1^{(A1)} & \theta_2^{(A1)} & \dots & \theta_i^{(B1)} & \dots & \theta_n^{(A1)} \\ \theta_1^{(A2)} & \theta_2^{(A2)} & \dots & \theta_i^{(B2)} & \dots & \theta_n^{(A2)} \\ \vdots & \vdots & \dots & \vdots & \dots & \vdots \\ \theta_1^{(AN)} & \theta_2^{(AN)} & \dots & \theta_i^{(BN)} & \dots & \theta_n^{(AN)} \end{pmatrix}$$

In the matrices A, B and A_B^i, each row represents a set of parameter to be used as an input for the model. Numerical simulations are performed, and the output of the sample matrices A, B and A_B^i are stored as the vectors

$$Y_A = \begin{pmatrix} Y\left(A^{(A1)}\right) \\ Y\left(A^{(A2)}\right) \\ \vdots \\ Y\left(A^{(AN)}\right) \end{pmatrix}; \quad Y_B = \begin{pmatrix} Y\left(B^{(B1)}\right) \\ Y\left(B^{(B2)}\right) \\ \vdots \\ Y\left(B^{(BN)}\right) \end{pmatrix}; \quad Y_{A_B^i}. \tag{5}$$

where Y_A, Y_B and $Y_{A_B^i}$ are output vectors.

The final step is to calculate the sensitivity indices, using the samples generated from the sampling scheme, described below. In this work, we are interested in computing the total effect indices, given by

$$S_{T_i} = 1 - \frac{Y_A \cdot Y_B - f^2}{Y_A \cdot Y_A - f^2} \tag{6}$$

where f is defined as

$$f := \frac{1}{N} \sum_{j=1}^{N} Y_A^{(j)}. \tag{7}$$

The total effect index indicates the contribution of the parameter to the output of the model. The importance of the parameter is proportional to the value of S_{T_i}, meaning that higher S_{T_i} leads to a higher contribution to the model output[2]. Parameters with higher S_T need a more carefully calibration, as small error during the calibration can lead to bigger errors to the model prediction. Of note, the total effect takes into account higher-order interactions among model variables; thus, correlation between variables can be identified by this method. We also evaluated the influence of first-order effects, which do not consider interactions among variables, to the model output.

In Figure 1, the result of the sensitivity analysis over time is presented. The numerical simulations were performed using SALib library[3]. The experiment was conducted generating $N = 12{,}000$ parameter combinations, totaling 120,000 simulations of the model, and the result shows the evolution of the parameters according to I_a, I_s, U, H and D compartments. We compared the results obtained using the total effect index (Figure 1 a,c,e,g,i) with those of the first-order effects, S_i (Figure 1 b,d,f,h,j), which measures the amount of variance in results that can be attributed to parameter i alone. Thus, in this model, the S_i is not able to adequately capture the influence of individual parameters due to the extensive non-linearity of the SEIIHURD model. For this reason, the total effect index was prominently used to evaluate overall influence of the model parameters on the SEIIHURD dynamics.

The results of the sensitivity analysis indicate that the factor that reduces the asymptomatic infectivity, δ, is among the most influential parameters to every model output during most of the period evaluated (i.e., 70 days). The analysis also indicates that during the first 30 days β_0 is the most important parameters in the system, as indicated by higher values of S_T. After this period, the importance of β_0 decreases, and β_1 becomes the most important parameter in the system, which is expected given the change in transmission rate occurring in this timepoint. For H, U, and D, the most influential parameter during the initial stages of the simulation (i.e., before day 15) is the proportion of symptomatic needing hospitalization or ICU, h. From these results we are able to conclude that a careful characterization of the parameters δ, β_0, β_1, and h is needed. Finally, the sensitivity index of the k parameter in all cases is close to zero, which means that it can be set fixed.

We also performed a snapshot comparison of model parameters in selected days at the beginning of the simulation (day 10), at a mid-point when a change in the transmission rate occurs ($\beta_0 \rightarrow \beta_1$; around day 25) and in a final simulation point when the influence of parameters present a greater stabilization (day 60) (Figure 2). These results reinforce the importance of δ to the dynamics of the system across all compartments in most of the detailed periods.

Supplementary Note 3
Case-clustering effect analysis

Case-clustering effect analysis

In order to evaluate possible case-clustering effects on our results, additional analyses were conducted by considering that the number of infections, deaths and clinical/ICU bed requirements, up to May 4, were all clustered in the city of Salvador, rather than scattered throughout the whole state of Bahia. Under this scenario, we fitted our model to the data using the population of 2,872,347 inhabitants, corresponding to the population size of Salvador. The estimated and fixed parameters, for the purpose of simulations here, are given in the following table:

Table 1: Key epidemiological parameters used in the SEIIHURD model for the case-clustering effect analysis.

Parameter	Description	Values (IC when estimated)
β_0	Pre-intervention transmission rate	1.27, (1.25; 1.28)
β_1	Post-intervention transmission rate	0.92, (0.91; 0.93)
t_1	Time of transmission rate change	April 9
δ	Factor that reduces the asymptomatic infectivity	0.34, (0.33, 0.35)
p	Proportion of latent (E) that proceed to symptomatic infective	0.2
κ	Mean exposed period	1/4
γ_a	Mean asymptomatic period	1/3.5
γ_s	Mean symptomatic period	1/4
h	Proportion of symptomatic needing hospitalization or ICU	0.28, (0.27, 0.28)
$1-\xi$	Proportion of symptomatic that proceed to ICU	0.47
γ_H	Mean hospitalization (clinical beds) period	0.13, (0.13; 0.14)
γ_U	Mean ICU period	0.14, (0.13, 0.14)
μ_H	Death rate of hospitalized individuals	0.15
μ_U	Death rate of ICU individuals	0.4
ω_H	Proportion of hospitalized that goes to ICU	0.14
ω_U	Proportion of ICU that goes to hospitalization	0.29

We estimated an initial transmission rate β_0 with a decrease to β_1 on April 9 as shown in Table 1, above. Under these conditions, predictions of the collapse of heath care systems in the absence of intervention would take place on April 25 and April 26 for clinical and UCI beds, respectively. With the maintenance of the current level of interventions determined by β_1, this depletion is shifted in time and would occur by May 3 and May 7, respectively. Comparing the previous result with the analysis considering the whole state (described in the main manuscript text), we can see that the collapses without intervention coincide with each other. However, under the maintenance of the second reduction, the predicted date of collapse occurs earlier when we consider the cluster in Salvador.

To see the scenario of future interventions, we present in Table 2 (below) the effects of the intensity and required intervention duration in order to delay the collapse of the system, when actions are enforced on May 2. Note that since the predicted collapse of clinical bed occurs on May 3, any effort on May 2 will be insufficient to change this condition. Nevertheless, we can evaluate the impact on ICU beds, for which the collapse is predicted to occur five days later. In this case, we see that the qualitative behaviour of our model remains unaltered. Nevertheless, interventions permit a postponing of the collapse for a much greater time interval.

Table 2: Scenarios of an immediate intervention in May 2, 2020, with variations of the transmission rate and intervention length.

Percentage of transmission rate reduction	Intervention length (days)	Date of hospitalization beds collapse (delay, in days, compared to baseline scenario)	Date of ICU beds collapse (delay, in days, compared to baseline scenario)
	7	05/03/20 (0)	05/08/20 (1)
25%	14	05/03/20 (0)	05/08/20 (1)
	30	05/03/20 (0)	05/08/20 (1)
	7	05/03/20 (0)	05/11/20 (4)
50%	14	05/03/20 (0)	05/11/20 (4)
	30	05/03/20 (0)	05/11/20 (4)
	7	05/03/20 (0)	05/19/20 (12)
75%	14	05/03/20 (0)	06/08/20 (32)
	30	05/03/20 (0)	07/17/20 (71)

It is relevant to emphasize that the administrative distribution of clinical and ICU beds in Brazil does not depend on each city's administration. Rather, management is centralized at a higher level within each Brazilian state, and are administratively referred as Health Regions (in portuguese, Regiões de Saúde). These Health Regions are planned with the aim of optimizing the management of the health system, the rationalization of resources and the institutional contribution to the creation of health care networks[30]. The access and use of health services are related to the provision of these services and imply patterns of user flows in the territories for sharing the health care network. Therefore, an infected person in a city (or even cluster) Y may be directed to a less affected city (or cluster) X.

As pointed in the Discussion, heterogeneous approaches (involving e.g. metapopulation models) are more appropriate to fully capture and describe the effects of case-clustering.

Supplementary Note 4
Differential Equations extended

Differential Equations extended

The extended modelling process of the system of differential equations (2) to (9) in the main manuscript is given as follow.

$$
\begin{aligned}
\frac{dS}{dt} &= \frac{-\beta(t)S(I_s+\delta I_a)}{N} && (8)\\
\frac{dE}{dt} &= \frac{\beta(t)S(I_s+\delta I_a)}{N} - \kappa E && (9)\\
\frac{dI_a}{dt} &= (1-p)\kappa E - \gamma_a I_a && (10)\\
\frac{dI_s}{dt} &= p\kappa E - \underbrace{(1-h)\gamma_s I_s}_{\text{recover: no need of H or U}} - \underbrace{h\xi\gamma_s I_s}_{\text{go to H}} - \underbrace{h(1-\xi)\gamma_s I_s}_{\text{go to U}} && (11)\\
\frac{dH}{dt} &= h\xi\gamma_s I_s + \underbrace{\omega_U\gamma_U U}_{\text{came from U}} + \underbrace{(1-\omega_U)(1-\mu_U)\gamma_U U}_{\text{came from U}} - \underbrace{\omega_H\gamma_H H}_{\text{go to U}} - \underbrace{(1-\omega_H)(1-\mu_H)\gamma_H H}_{\text{recover}} - \underbrace{(1-\omega_H)\mu_H\gamma_H H}_{\text{Die}} && (12)\\
\frac{dU}{dt} &= h(1-\xi)\gamma_s I_s + \underbrace{\omega_H\gamma_H H}_{\text{came from H}} - \underbrace{\omega_U\gamma_U U}_{\text{go to H}} - \underbrace{(1-\omega_U)\mu_U\gamma_U U}_{\text{die}} - \underbrace{(1-\omega_U)(1-\mu_U)\gamma_U U}_{\text{Came back to H before recover}} && (13)\\
\frac{dR}{dt} &= \gamma_a I_a + (1-h)\gamma_s I_s + (1-\mu_H)(1-\omega_H)\gamma_H H && (14)\\
\frac{dD}{dt} &= (1-\omega_H)\mu_H\gamma_H H + (1-\omega_U)\mu_U\gamma_U U && (15)
\end{aligned}
$$

Simplifying, Eq(11) becomes:

$$
\begin{aligned}
-(1-h)\gamma_s I_s - h\xi\gamma_s I_s - h(1-\xi)\gamma_s I_s &= -(1-h)\gamma_s I_s - h\xi\gamma_s I_s - h\gamma_s I_s + h\xi\gamma_s I_s && (16)\\
&= -(1-h)\gamma_s I_s - h\gamma_s I_s && (17)\\
&= \gamma_s I_s. && (18)
\end{aligned}
$$

Therefore $\frac{dI_s}{dt} = p\kappa E - \gamma_s I_s$.
In Eq(12) we have:

$$
\begin{aligned}
&\omega_U\gamma_U U + (1-\omega_U)(1-\mu_U)\gamma_U U - \omega_H\gamma_H H - (1-\omega_H)(1-\mu_H)\gamma_H H - (1-\omega_H)\mu_H\gamma_H H && (19)\\
=&\omega_U\gamma_U U + (1-\omega_U)\gamma_U U - (1-\omega_U)\mu_U\gamma_U U - \omega_H\gamma_H H - (1-\omega_H)\gamma_H H + (1-\omega_H)\mu_H\gamma_H H - (1-\omega_H)\mu_H\gamma_H H && (20)\\
=&\omega_U\gamma_U U + \gamma_U U - \omega_U\gamma_U U - (1-\omega_U)\mu_U\gamma_U U - \omega_H\gamma_H H - \gamma_H H + \omega_H\gamma_H H && (21)\\
=&\gamma_U U - (1-\omega_U)\mu_U\gamma_U U - \gamma_H H && (22)\\
=&(1-\mu_U+\omega_U\mu_U)\gamma_U U - \gamma_H H. && (23)
\end{aligned}
$$

Thus, $\frac{dH}{dt} = h\xi\gamma_s I_s + (1-\mu_U+\omega_U\mu_U)\gamma_U U - \gamma_H H$.
To conclude, Eq(13) yields:

$$
\begin{aligned}
&-\omega_U\gamma_U U - (1-\omega_U)\mu_U\gamma_U U - (1-\omega_U)(1-\mu_U)\gamma_U U && (24)\\
=&-\omega_U\gamma_U U - (1-\omega_U)\mu_U\gamma_U U - (1-\omega_U)\gamma_U U + (1-\omega_U)\mu_U\gamma_U U && (25)\\
=&-\omega_U\gamma_U U - (1-\omega_U)\gamma_U U && (26)\\
=&-\gamma_U U. && (27)
\end{aligned}
$$

Thus, $\frac{dU}{dt} = h(1-\xi)\gamma_s I_s + \omega_H\gamma_H H - \gamma_U U$.

Supplementary Note 5
Derivation of the effective reproduction number

Derivation of the effective reproduction number

Different approaches have been used to estimate the effective reproduction number in the course of a pandemic, most of them relying on the daily count of new cases, the basic available information to analyse the course of an emerging disease.

Here we present a derivation of the renewal equation for the SEIIHURD model. The renewal equation is often used to study the age-structured population growth. It was first used within the scope of mathematical demography with the aim to obtain the total number of female offspring produced by a mother over her lifespan. As an analogy, based on the series of daily infected individuals, we can apply the same methodology to obtain the average number of secondary infections that an individual, who became infected at time τ, is able to generate[24].

$J(t)$, the rate of new infections at time t caused by individuals infected at a previews time $\tau \leq t$, is equal to the number of new infections at time $t-\tau$ multiplied by the expected number of new infections $A(t,\tau)$ an individual will generate. Summing up these new infections over all individuals infected at any time, we obtain that the total number of new infections at time t can be expressed by

$$J(t) = \int_{\tau=0}^{\infty} J(t-\tau)A(t,\tau)d\tau, \tag{28}$$

which is known as the renewal equation. The quantity $A(t,\tau)$ is referred to as the rate of new infections an infected individual produces through time, whereby $A(t,\tau > t) \equiv 0$. Thus, if we integrate $A(t,\tau)$ over an interval of time $[0,\infty]$, we obtain the total number of secondary infections expected by that individual during the infectious time, given by:

$$\mathscr{R}(t) = \int_{\tau=0}^{\infty} A(t,\tau)d\tau. \tag{29}$$

The SEIIHURD model belongs to a wide class of models for which $A(t,\tau)$ can be written as the product of two general separate functions of t and τ, i.e., $A(t,\tau) = \phi_1(t)\phi_2(\tau)$, which in the present case can be identified with $\mathscr{R}(t)$ and $g(\tau)$[24,25]. Indeed, after choosing a normalized $g(\tau)$ function

$$\int_{0}^{\infty} g(\tau)\, d\tau \equiv 1, \tag{30}$$

it follows from (29) that $\phi_1 = \mathscr{R}(t)$. Therefore, from (29) we get

$$g(\tau) = \frac{A(t,\tau)}{\int_{\tau=0}^{\infty} A(t,\tau)d\tau}, \tag{31}$$

which leads to

$$A(t,\tau) = \mathscr{R}(t)g(\tau). \tag{32}$$

After substituting (32) in Equation (28), we obtain

$$J(t) = \mathscr{R}(t)\int_{\tau=0}^{\infty} J(t-\tau)g(\tau)d\tau, \tag{33}$$

so that the general expression for $\mathscr{R}(t)$ can be written as

$$\mathscr{R}(t) = \frac{J(t)}{\int_{\tau=0}^{\infty} J(t-\tau)g(\tau)d\tau}. \tag{34}$$

To obtain the renewal equation and, consequently, the reproduction number for the SEIIHURD model, we consider an analogous infection-age structured model. The infection-age models considers not only the dynamics of the disease transmission but also the time elapsed since the infection. Those models, which are derived from the population growth structured model, are the origins for the widely known Kermack-Mckendrick ODE's models (SIR and SEIR). The system of equations for the susceptible, exposed, symptomatic and asymptomatic individuals for the SEIIHURD infection-age model can be written as

$$\frac{dS}{dt} = -\frac{\beta}{N}\,S(t)\,\left(I_s(t) + \delta I_a(t)\right), \tag{35}$$

$$\left(\frac{\partial}{\partial t} + \frac{\partial}{\partial \tau}\right) e(t,\tau) = -\,\kappa e(t,\tau), \tag{36}$$

$$e(t,\tau = 0) = \frac{\beta}{N}\,S(t)\,\left(I_s(t) + \delta I_a(t)\right)\; = J(t), \tag{37}$$

$$\left(\frac{\partial}{\partial t} + \frac{\partial}{\partial \tau}\right) i_a(t,\tau) = (1-p)\kappa\, e(t,\tau) - \gamma_a i_a(t,\tau), \tag{38}$$

$$\left(\frac{\partial}{\partial t} + \frac{\partial}{\partial \tau}\right) i_s(t,\tau) = p\kappa\, e(t,\tau) - \gamma_s i_s(t,\tau), \tag{39}$$

$$i_s(t,\tau = 0) = i_a(t,\tau = 0) = 0\,. \tag{40}$$

As in equation 28, τ indicates the infection-age, the time elapsed since the individual was infected, and t is the calendar time. γ_a and γ_s are the rate of recover and $J(t)$ the number of new infections, which is equivalent to the $e(t,\tau = 0)$ initial condition. As we will conclude at the end of this derivation, can be related to the reported cases $b(t)$. $e(t,\tau)$, $i_a(t,\tau)$ and $i_s(t,\tau)$ are the density of individuals in the exposed, asymptomatic and symptomatic compartments, respectively, for each infection-age at a specific instant of the calendar time. The total number of individuals in those compartments ($E(t)$, $I_a(t)$ and $I_s(t)$) are given by integrating the densities from zero to infinity in respect to τ.

Equations (36) to (39) can be solved by the method of integration along the characteristic line[25]. From the left side of the equations, we can see that the characteristics of those PDE's are lines of slope 1, which implies that $t = \tau + c$ with c being an arbitrary constant. To carry out the integration along the characteristic line, we fix a point (t_0,τ_0) and introduce a variable s such that $u(s) = e(t_0 + s, \tau_0 + s)$, $v_a(s) = i_a(t_0 + s, \tau_0 + s)$ and $v_s(s) = v_s(t_0 + s, \tau_0 + s)$ are functions that provides the values of the compartment densities along the characteristic line. Then, the derivatives through the characteristic can be operated as simply as

$$\frac{d}{ds} = \left(\frac{\partial}{\partial t} + \frac{\partial}{\partial \tau}\right). \tag{41}$$

The three functions of s must then obey the ODE system

$$\frac{du(s)}{ds} = -\,\kappa u(s), \tag{42}$$

$$\frac{dv_a(s)}{ds} = (1-p)\kappa u(s) - \gamma_a v_a(s), \tag{43}$$

$$\frac{dv_s(s)}{ds} = p\kappa u(s) - \gamma_s v_s(s). \tag{44}$$

We want to solve this system for $t_0 > \tau_0$, since there are no infected individuals prior to $t = 0$. Then, setting $\tau_0 = 0$ we have that $\tau = s$ and $t_0 = t - \tau$. This approach is then analogous to the one used in[26] for the SEIR model, resulting in the solutions for the density compartments given by

$$e(t,\tau) = J(t-\tau)e^{-k\tau}, \tag{45}$$

$$i_a(t,\tau) = J(t-\tau)(1-p)\frac{\kappa}{\kappa-\gamma_a}(e^{-\gamma_a\tau} - e^{-\kappa\tau}), \tag{46}$$

$$i_s(t,\tau) = J(t-\tau)p\frac{\kappa}{\kappa-\gamma_s}(e^{-\gamma_s\tau} - e^{-\kappa\tau}). \tag{47}$$

It is noteworthy to observe that if κ is equal to γ_s or γ_a, the corresponding expression for $i_x(\tau)$ should be replaced by taking the limit $\kappa \to \gamma_x$, which results in $J(t-\tau)\gamma_x\tau\, e^{-\gamma_x\tau}$ multiplied p or $(1-p)$. We can now insert equations (46) and (47) into (37) by integrating those densities over τ obtaining

$$J(t) = \frac{\beta}{N}\,S(t)\,\left(\int_0^\infty p\frac{\kappa}{\kappa-\gamma_s}(e^{-\gamma_s\tau} - e^{-\kappa\tau})J(t-\tau)d\tau + \delta\int_0^\infty (1-p)\frac{\kappa}{\kappa-\gamma_a}(e^{-\gamma_a\tau} - e^{-\kappa\tau})J(t-\tau)d\tau\right), \tag{48}$$

which is the renewal equation (28) of the SEIIHURD model, with

$$A_a(t,\tau) = \delta\frac{\beta}{N}S(t)(1-p)\ \frac{\kappa}{\kappa-\gamma_a}(e^{-\gamma_a\tau}-e^{-\kappa\tau}), \tag{49}$$

$$A_s(t,\tau) = \frac{\beta}{N}S(t)p\ \frac{\kappa}{\kappa-\gamma_s}(e^{-\gamma_s\tau}-e^{-\kappa\tau}), \tag{50}$$

$$A(t,\tau) = A_a(t,\tau)+A_s(t,\tau). \tag{51}$$

Thus, the $\mathscr{R}(t)$ of this model can be obtained as in (29):

$$\begin{aligned}\mathscr{R}(t) &= \int_0^\infty A(t,\tau)d\tau \\ &= \int_0^\infty A_a(t,\tau)d\tau + \int_0^\infty A_s(t,\tau)d\tau \\ &= \frac{S(t)}{S(0)}\frac{\delta(1-p)\beta}{\gamma_a} + \frac{S(t)}{S(0)}\frac{p\beta}{\gamma_s} \\ &= \frac{S(t)}{S(0)}\mathscr{R}_{a0} + \frac{S(t)}{S(0)}\mathscr{R}_{s0}\end{aligned} \tag{52}$$

The reproduction number of the model decouples in two reproduction numbers, $\mathscr{R}_a(t) = \frac{S(t)}{S(0)}\mathscr{R}_{a0}$ and $\mathscr{R}_s(t) = \frac{S(t)}{S(0)}\mathscr{R}_{s0}$, representing the asymptomatic and the symptomatic reproduction numbers, respectively. $\mathscr{R}_{a0}$ and $\mathscr{R}_{s0}$ correspond to the basic reproduction numbers encountered with the next-generation method. Equation (52) reduces to the basic reproduction number for a calendar time equal zero. Therefore, the explicit form of the function $g(\tau)$ that follows from (31) is

$$g(\tau) = \frac{p\ g_s(\tau)/\gamma_s + \delta(1-p)\ g_a(\tau)/\gamma_a}{p/\gamma_s + \delta(1-p)/\gamma_a}, \tag{53}$$

where

$$g_a(\tau) = \frac{A_a(t,\tau)}{\int_{\tau=0}^{\infty}A_a(t,\tau)d\tau} = \frac{\kappa\gamma_a}{\kappa-\gamma_a}(e^{-\gamma_a\tau}-e^{-\kappa\tau}), \tag{54}$$

$$g_s(\tau) = \frac{A_s(t,\tau)}{\int_{\tau=0}^{\infty}A_s(t,\tau)d\tau} = \frac{\kappa\gamma_s}{\kappa-\gamma_s}(e^{-\gamma_s\tau}-e^{-\kappa\tau}). \tag{55}$$

The generation intervals of the asymptomatic and symptomatic individuals ($g_a(\tau)$ and $g_s(\tau)$) are the well known generation interval distribution for the SEIR model[27,28]. This expression takes into account the sequential contributions of flow $E\to I_a\to O$ and $E\to I_s\to O$, where O indicates any compartment not responsible for infectious steps. We can correlate equations (54) and (55) to the $\mathscr{T}$ matrix, found in the main text (11), following the Theorem 2.1 in M. Akkouchi (2008)[28] for the sub-models (E,I_a) and (E,I_s) given by

$$g_a(\tau) = (\prod_{i=1,2}\mathscr{T}_{ii})\sum_{i=1,2}\frac{\exp(-\mathscr{T}_{ii}\tau)}{\prod_{j=1,2,j\neq i}(\mathscr{T}_{jj}-\mathscr{T}_{ii})} = \frac{\kappa\gamma_a}{\kappa-\gamma_a}(e^{-\gamma_a\tau}-e^{-\kappa\tau}), \tag{56}$$

$$g_s(\tau) = (\prod_{i=1,3}\mathscr{T}_{ii})\sum_{i=1,3}\frac{\exp(-\mathscr{T}_{ii}\tau)}{\prod_{j=1,3,j\neq i}(\mathscr{T}_{jj}-\mathscr{T}_{ii})} = \frac{\kappa\gamma_s}{\kappa-\gamma_s}(e^{-\gamma_s\tau}-e^{-\kappa\tau}). \tag{57}$$

Since $J(t)$ is the rate of new infections, we can correlate it to the observed data. It's usually assumed that the cases are reported when the exposed individual becomes a infected symptomatic, so we need to consider the latent time between the infection and the observation. Also, in the SEIIHURD model we assume that a portion "p" of the exposed individuals will present symptoms. So the number of new reported cases for this model is $b(t) = p\,J(t-\ell)$, with $\ell = 1/\kappa$ being the average latent time. Thus, in the equation (34) for the $\mathscr{R}(t)$ we have $J(t) = b(t+\ell)/p$.

Supplementary Note 6

Identifiability analysis of the SEIIHURD model

Identifiability analysis of the SEIIHURD model

Given the complexity of the proposed model, in terms of the number of parameters and possible correlations between them, a simulation study was carried out in order to assess the identifiability of the model presented. The study was conducted based on the approach discussed in Roosa and Chowell (2019)[29], which employs the parametric bootstrap method to generate data from a system of dynamic equations, in order to quantify the uncertainty and access the identifiability of the indicators of the model worked on.

A total of 2,048 bootstrap samples were generated, considering a Poisson distribution for the error structure and the identifiability of the model was evaluated through graphical analysis[29]. Confidence intervals and the MSE were built for each parameter. This analysis considered the dimension of the parameter space Θ, since the larger the parameter space to be estimated, the greater the chances of the model being non-identifiable. Thus, the space Θ was divided into sub-spaces $\Theta_i,\ i = 1,\ldots,7$, where i corresponds to the maximum number of parameters being jointly estimated. Thus, Θ_1 considers the estimation of only 1 parameter, keeping the others fixed, and Θ_7 jointly estimates all parameters of the model. By increasing the number of parameters estimated together, it is possible to assess the impact of this increase on the variability of the parameter, thus evaluating a possible lack of identifiability.

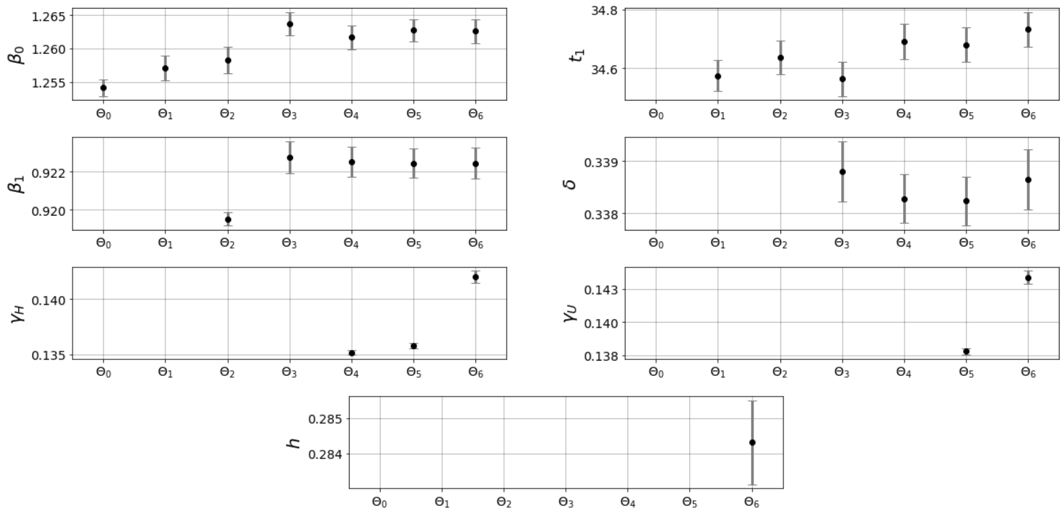

Identifiability analysis of the SEIIHURD model for Bahia. 95% confidence intervals for the distributions of each estimated parameter obtained from the 2,048 realizations of the simulated datasets.

In the figure above, all parameters, except t_1, δ, and h, have accurate confidence intervals in all sub-spaces considered. However, when more parameters are jointly estimated, the point estimates for β_1, β_2, t_1, γ_H, and γ_U are higher. As described in Roosa and Chowell (2019)[29], "small confidence intervals with a finite range of values may indicate that the parameter can be precisely identified, while a wider range could be indicative of a lack of identifiability". The MSE for each estimated parameter is presented in the figure below. A higher MSE is observed for δ, γ_U and γ_H when more than five parameters are jointly estimated. These results suggest that parameter non-identifiability can occur when more than five parameters are jointly estimated. Nevertheless, the MSE are of the order of 10^{-2}, an improvement that is gained with the inclusion of more data to inform the mathematical model.

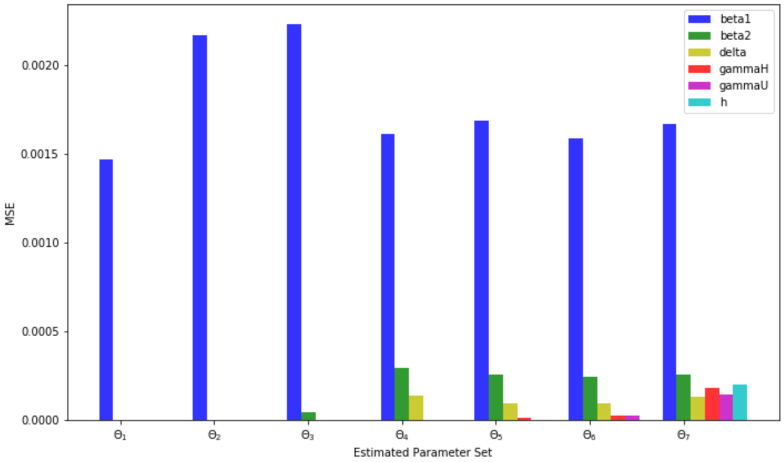

Mean squared error (MSE) of the distribution of parameter of the identifiability analysis. Estimates (2,048 realizations) for each estimated parameter were used.

Supplementary References

1. Sobol, Ilya M Global sensitivity indices for nonlinear mathematical models and their Monte–Carlo estimates. Mathematics and Computers in Simulation, 55, 1-3, 271–280, (2001).

2. Saltelli, Andrea and Ratto, Marco and Andres, Terry and Campolongo, Francesca and Cariboni, Jessica and Gatelli, Debora and Saisana, Michaela and Tarantola, Stefano. Global Sensitivity Analysis: The Primer. John Wiley & Sons, (2008).

3. Jon Herman and Will Usher. SALib: An open-source Python library for Sensitivity Analysis. The Journal of Open Source Software, 2, 9, (2017).

4. Instituto Brasileiro de Geografia e Estatística - IBGE. In: Estimates for the population of Bahia in 2019 [Internet]. [cited 1 Mar 2020]. Available: https://cidades.ibge.gov.br/brasil/ba/panorama.

5. Lin Q, Zhao S, Gao D, Lou Y, Yang S, Musa SS, et al. A conceptual model for the coronavirus disease 2019 (COVID-19) outbreak in Wuhan, China with individual reaction and governmental action. Int J Infect Dis., 93: 211–216, (2020).

6. Li, Ruiyun and Pei, Sen and Chen, Bin and Song, Yimeng and Zhang, Tao and Yang, Wan and Shaman, Jeffrey. Substantial undocumented infection facilitates the rapid dissemination of novel coronavirus (SARS-CoV-2). Science, 368, 6490, 489–493, (2020).

7. Ferretti L, Wymant C, Kendall M, Zhao L, Nurtay A, Abeler-Dörner L, et al. Quantifying SARS-CoV-2 transmission suggests epidemic control with digital contact tracing. Science., doi:10.1126/science.abb6936, (2020).

8. Ferguson, Neil and Laydon, Daniel and Nedjati Gilani, Gemma and Imai, Natsuko and Ainslie, Kylie and Baguelin, Marc and Bhatia, Sangeeta and Boonyasiri, Adhiratha and Cucunuba Perez, ZULMA and Cuomo-Dannenburg, Gina and others. Impact of non-pharmaceutical interventions (NPIs) to reduce COVID19 mortality and healthcare demand. pre-print available at: http://hdl.handle.net/10044/1/77482, (2020)

9. Kissler, Stephen M and Tedijanto, Christine and Goldstein, Edward and Grad, Yonatan H and Lipsitch, Marc. Projecting the transmission dynamics of SARS-CoV-2 through the postpandemic period. Science, 368, 6493, 860-868, (2020).

10. Li, Qun and Guan, Xuhua and Wu, Peng and Wang, Xiaoye and Zhou, Lei and Tong, Yeqing and Ren, Ruiqi and Leung, Kathy SM and Lau, Eric HY and Wong, Jessica Y and others. Early transmission dynamics in Wuhan, China, of novel coronavirus–infected pneumonia. New England Journal of Medicine, 382:1199-1207, (2020).

11. Backer JA, Klinkenberg D, Wallinga J. Incubation period of 2019 novel coronavirus (2019-nCoV) infections among travellers from Wuhan, China, 20-28 January 2020. Euro Surveill. 25. doi:10.2807/1560-7917.ES.2020.25.5.2000062, (2020).

12. Linton NM, Kobayashi T, Yang Y, Hayashi K, Akhmetzhanov AR, Jung S-M, et al. Incubation Period and Other Epidemiological Characteristics of 2019 Novel Coronavirus Infections with Right Truncation: A Statistical Analysis of Publicly Available Case Data. J Clin Med Res. 9. doi:10.3390/jcm9020538, (2020).

13. Sanche S, Lin YT, Xu C, Romero-Severson E, Hengartner N, Ke R. High Contagiousness and Rapid Spread of Severe Acute Respiratory Syndrome Coronavirus 2. Emerg Infect Dis. 26. doi:10.3201/eid2607.200282, (2020).

14. Lauer SA, Grantz KH, Bi Q, Jones FK, Zheng Q, Meredith HR, et al. The Incubation Period of Coronavirus Disease 2019 (COVID-19) From Publicly Reported Confirmed Cases: Estimation and Application. Ann Intern Med. doi:10.7326/M20-0504, (2020).

15. Guan W-J, Ni Z-Y, Hu Y, Liang W-H, Ou C-Q, He J-X, et al. Clinical Characteristics of Coronavirus Disease 2019 in China. N Engl J Med. doi:10.1056/NEJMoa2002032, (2020).

16. Mizumoto K, Kagaya K, Zarebski A, Chowell G. Estimating the asymptomatic proportion of coronavirus disease 2019 (COVID-19) cases on board the Diamond Princess cruise ship, Yokohama, Japan, 2020. Euro Surveill. 25. doi:10.2807/1560-7917.ES.2020.25.10.2000180, (2020).

17. Nishiura H, Kobayashi T, Suzuki A, Jung S-M, Hayashi K, Kinoshita R, et al. Estimation of the asymptomatic ratio of novel coronavirus infections (COVID-19). Int J Infect Dis. doi:10.1016/j.ijid.2020.03.020, (2020).

18. Castro, Marcia C and de Carvalho, Lucas Resende and Chin, Taylor and Kahn, Rebecca and Franca, Giovanny VA and Macario, Eduardo Marques and de Oliveira, Wanderson Kleber. Demand for hospitalization services for COVID-19 patients in Brazil. Preprint at https://doi.org/10.1101/2020.03.30.20047662, (2020).

19. Wu Z, McGoogan JM. Characteristics of and Important Lessons From the Coronavirus Disease 2019 (COVID-19) Outbreak in China: Summary of a Report of 72 314 Cases From the Chinese Center for Disease Control and Prevention. JAMA. doi:10.1001/jama.2020.2648, (2020).

20. Zhou F, Yu T, Du R, Fan G, Liu Y, Liu Z, et al. Clinical course and risk factors for mortality of adult inpatients with COVID-19 in Wuhan, China: a retrospective cohort study. Lancet. 395: 1054–1062, (2020) .

21. Wang D, Hu B, Hu C, Zhu F, Liu X, Zhang J, et al. Clinical Characteristics of 138 Hospitalized Patients With 2019 Novel Coronavirus-Infected Pneumonia in Wuhan, China. JAMA. doi:10.1001/jama.2020.1585, (2020).

22. Chen J, Qi T, Liu L, Ling Y, Qian Z, Li T, et al. Clinical progression of patients with COVID-19 in Shanghai, China. J Infect. doi:10.1016/j.jinf.2020.03.004, (2020).

23. Petrilli CM, Jones SA, Yang J, Rajagopalan H, O'Donnell LF, Chernyak Y, et al. Factors associated with hospitalization and critical illness among 4,103 patients with COVID-19 disease in New York City. Intensive Care and Critical Care Medicine. medRxiv; 2020.

24. Wallinga, Jacco and Lipsitch, Marc. How generation intervals shape the relationship between growth rates and reproductive numbers. Proceedings of the Royal Society B: Biological Sciences, 274, 1609, 599–604, (2007).

25. Nishiura, Hiroshi and Chowell, Gerardo. The effective reproduction number as a prelude to statistical estimation of time-dependent epidemic trends. Mathematical and statistical estimation approaches in epidemiology, 103–121, (2009).

26. Brauer, Fred Age of infection in epidemiology models. Electronic Journal of Differential Equations (EJDE), 2005,29–37, (2005).

27. Champredon, David and Dushoff, Jonathan and Earn, David JD Equivalence of the Erlang-Distributed SEIR Epidemic Model and the Renewal Equation. SIAM J. Appl. Math., 78(6), 3258–3278, (2018).

28. Akkouchi, Mohamed On the convolution of exponential distributions. J. Chungcheong Math. Soc, 21, 4, 501–510,(2008).

29. Roosa, Kimberlyn and Chowell, Gerardo. Assessing parameter identifiability in compartmental dynamic models using a computational approach: application to infectious disease transmission models. Theoretical Biology and Medical Modelling, 16, 1, 1,(2019).

30. Xavier, Diego Ricardo, Oliveira, Ricardo Antunes Dantas de, Barcellos, Christovam, Saldanha, Raphael de Freitas, Ramalho, Walter Massa, Laguardia, Josué, Viacava, Francisco. As Regiões de Saúde no Brasil segundo internações: método para apoio na regionalização de saúde. Cadernos de Saúde Pública, 35(Suppl. 2), e00076118. (2019).
