# Supplementary figures and images for: Mathematical modeling of COVID-19 in 14.8 million individuals in Bahia, Brazil
Source: Nat Commun. 2021 Jan 12;12:333. doi: 10.1038/s41467-020-19798-3 (PMC7803757; doi:10.1038/s41467-020-19798-3)

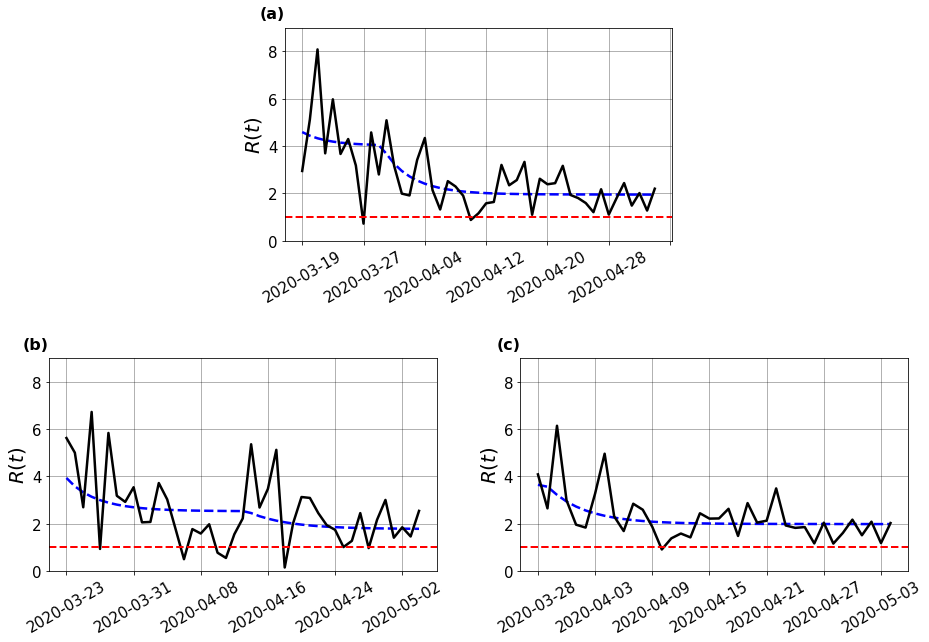

Supplement: Supplementary file 3 — Source Data [file 41467_2020_19798_MOESM3_ESM.zip › Supplementary Material/Effective reproduction number/Figs//BA.png]

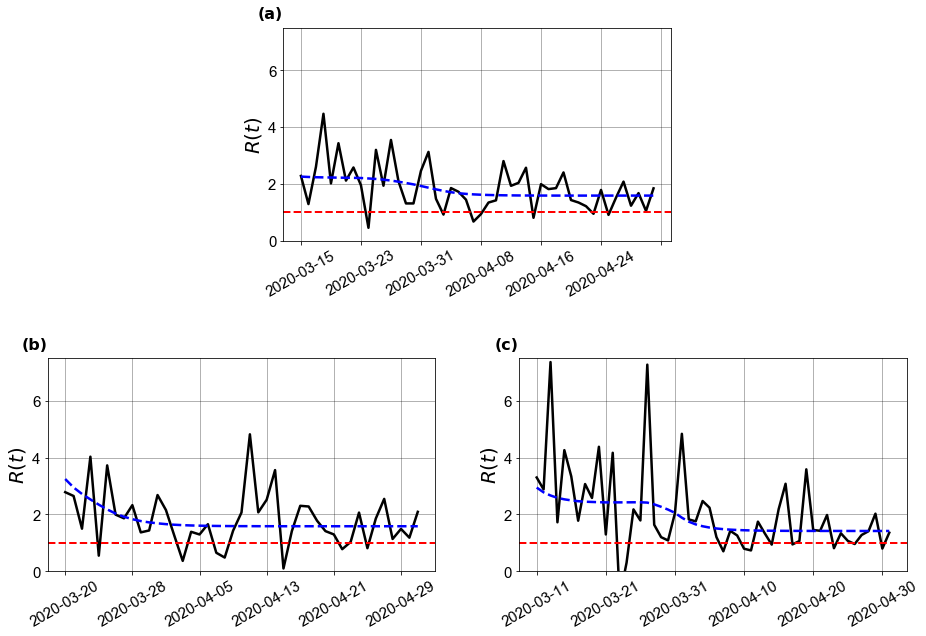

Supplement: Supplementary file 3 — Source Data [file 41467_2020_19798_MOESM3_ESM.zip › Supplementary Material/Effective reproduction number/Rt_BA.png]

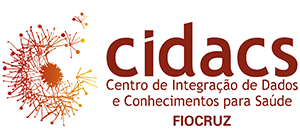

Supplement: Supplementary file 3 — Source Data [file 41467_2020_19798_MOESM3_ESM.zip › Supplementary Material/images/cidacs.png]
